# Expression Changes Confirm Genomic Variants Predicted to Result in Allele-Specific, Alternative mRNA Splicing

**DOI:** 10.3389/fgene.2020.00109

**Published:** 2020-03-05

**Authors:** Eliseos J. Mucaki, Ben C. Shirley, Peter K. Rogan

**Affiliations:** ^1^Department of Biochemistry, University of Western Ontario, London, ON, Canada; ^2^CytoGnomix, London, ON, Canada; ^3^Department of Oncology University of Western Ontario, London, ON, Canada; ^4^Department of Computer Science, University of Western Ontario, London, ON, Canada

**Keywords:** allele-specific gene expression, mRNA splicing, single nucleotide polymorphism, mutation, cryptic splicing, intron retention, alternative splicing, information theory

## Abstract

Splice isoform structure and abundance can be affected by either noncoding or masquerading coding variants that alter the structure or abundance of transcripts. When these variants are common in the population, these nonconstitutive transcripts are sufficiently frequent so as to resemble naturally occurring, alternative mRNA splicing. Prediction of the effects of such variants has been shown to be accurate using information theory-based methods. Single nucleotide polymorphisms (SNPs) predicted to significantly alter natural and/or cryptic splice site strength were shown to affect gene expression. Splicing changes for known SNP genotypes were confirmed in HapMap lymphoblastoid cell lines with gene expression microarrays and custom designed q-RT-PCR or TaqMan assays. The majority of these SNPs (15 of 22) as well as an independent set of 24 variants were then subjected to RNAseq analysis using the ValidSpliceMut web beacon (http://validsplicemut.cytognomix.com), which is based on data from the Cancer Genome Atlas and International Cancer Genome Consortium. SNPs from different genes analyzed with gene expression microarray and q-RT-PCR exhibited significant changes in affected splice site use. Thirteen SNPs directly affected exon inclusion and 10 altered cryptic site use. Homozygous SNP genotypes resulting in stronger splice sites exhibited higher levels of processed mRNA than alleles associated with weaker sites. Four SNPs exhibited variable expression among individuals with the same genotypes, masking statistically significant expression differences between alleles. Genome-wide information theory and expression analyses (RNAseq) in tumor exomes and genomes confirmed splicing effects for 7 of the HapMap SNP and 14 SNPs identified from tumor genomes. q-RT-PCR resolved rare splice isoforms with read abundance too low for statistical significance in ValidSpliceMut. Nevertheless, the web-beacon provides evidence of unanticipated splicing outcomes, for example, intron retention due to compromised recognition of constitutive splice sites. Thus, ValidSpliceMut and q-RT-PCR represent complementary resources for identification of allele-specific, alternative splicing.

## Introduction

Accurate and comprehensive methods are needed for predicting impact of noncoding mutations, in particular, mRNA splicing defects, which are prevalent in genetic disease (Krawczak et al., 1992; Teraoka et al., 1999; Ars et al., 2003; Spielmann and Mundlos, 2016; Gloss and Dinger, 2018). This class of mutations may account for as much as 62% of point mutations (López-Bigas et al., 2005). Large transcriptome studies have suggested that a large fraction of genome-wide association studies (GWAS) signals for disease and complex traits are due to single nucleotide polymorphisms (SNPs) affecting mRNA splicing (Park et al., 2018). ValidSpliceMut (Shirley et al., 2019) presents evidence of altered splicing (Viner et al., 2014; Dorman et al., 2014) for 309,848 validated genome splice-variant predictions (Shirley et al., 2013). The majority of mutations were associated with exon skipping, cryptic site use, or intron retention, and in these cases ValidSpliceMut assigns a molecular phenotype classification to all variants as either aberrant, likely aberrant or inducing alternative isoforms.

While allele-specific alternative splicing can predispose for disease susceptibility (Park et al., 2018), these genetic variations also are associated with common phenotypic variability in populations (Hull et al., 2007). Soemedi et al. (2017) determined that 10% of a set of published disease-causing exonic mutations (N = 4,964) altered splicing. Their analysis of a control set of exonic SNPs common among those without disease phenotypes revealed a smaller proportion (3%) that altered splicing (N = 228). However, we recently showed that splice-altering, common SNPs are considerably more abundant in tumor genomes in the ValidSpliceMut web-beacon (http://validsplicemut.cytognomix.com; Shirley et al., 2019). Variants with higher germline population frequencies which impact splicing are less likely than rare mutations with direct splicing effects to be involved in Mendelian diseases or cancer. The present study analyzes predicted splice-altering polymorphic variants in genotyped lymphoblastoid cell lines by q-RT-PCR, expression microarrays of samples of known SNP genotypes, and high throughput expression data corresponding to sequenced tumor exomes and genomes. The relatively high frequencies of these variants enable comparisons of expressed transcripts in multiple individuals and genotypes. Effects of these SNPs are confirmed by multiple methods, although the supporting evidence from these distinct approaches is often complementary, rather than entirely concordant.

An estimated 90% to 95% of all multiexon genes are alternatively spliced (Pan et al., 2008; Wang et al., 2008; Baralle and Giudice, 2017). The selection of splicing signals involves exon and intron sequences, complementarity with snRNAs, RNA secondary structure, and competition between spliceosomal recognition sites (Moore and Sharp, 1993; Berget, 1995; Park et al., 2018). U1 snRNP interacts with the donor (or 5') splice site (Zhuang and Weiner, 1986; Séraphin et al., 1988) and U2 (and U6) snRNP with the acceptor and branch sites of pre-mRNA (Parker et al., 1987; Wu and Manley, 1989). The majority of human splice donors (5') and acceptors (3') base pair with the U1 and U2 RNAs in spliceosomes, but are generally not precisely complementary to these sequences (Rogan et al., 2003). Additional exonic and intronic cis-regulatory elements can promote or suppress splice site recognition through recruitment of trans-acting splicing factors. SR proteins are positive trans-acting splicing factors which contain RNA-recognition motifs (RRM) and a carboxy-terminal domain enriched in Arg/Ser dipeptides (SR domain; Birney et al., 1993). Binding of RRMs in pre-mRNA enhances exon recognition by promoting interactions with spliceosomal and other proteins (Fu and Maniatis, 1992). SR proteins function in splice site communication by forming an intron bridge needed for exon recognition (Zuo and Maniatis, 1996). Factors that negatively impact splicing include heterogeneous nuclear ribonucleoproteins (hnRNPs; Martinez-Contreras et al., 2007).

Splicing mutations affect normal exon recognition by altering the strengths of natural donor or acceptor sites and proximate cryptic sites, either independently or simultaneously. Weakened splice sites reduce of kinetics of mRNA processing, leading to an overall decrease in full length transcripts, increased exon skipping, cryptic splice site activation within exons or within adjacent introns, intron retention, and inclusion of cryptic, pseudo-exons (Talerico and Berget, 1990; Carothers et al., 1993; Buratti et al., 2006; Park et al., 2018). The kinetics of splicing at weaker cryptic sites is also slower than at natural sites (Domenjoud et al., 1993). Mutations strengthen cryptic sites either by increasing resemblance to “consensus sequences” (Nelson and Green, 1990) or by modulating the levels of SR proteins contributing to splice site recognition (Mayeda and Krainer, 1992; Cáceres et al., 1994). Mutations affecting splicing regulatory elements (Dietz et al., 1993; Richard and Beckmann, 1995) disrupt trans-acting SR protein interactions (Staknis and Reed, 1994) with distinct exonic and intronic cis-regulatory elements (Black, 2003).

Information theory-based (IT-based) models of donor and acceptor mRNA splice sites reveal the effects of changes in strengths of individual sites (termed *R_i_*; Rogan et al., 1998; Rogan et al., 2003). This facilitates prediction of phenotypic severity (Rogan and Schneider, 1995; von Kodolitsch et al., 1999; von Kodolitsch et al., 2006). The effects of splicing mutations can be predicted *in silico* by information theory (Rogan and Schneider, 1995; Kannabiran et al., 1998; Rogan et al., 1998; Svojanovsky et al., 2000; Rogan et al., 2003; Caminsky et al., 2014; Dorman et al., 2014; Viner et al., 2014; Caminsky et al., 2016; Mucaki et al., 2016; Shirley et al., 2019), and these predictions can be confirmed by *in vitro* experimental studies (Vockley et al., 2000; Lamba et al., 2003; Rogan et al., 2003; Khan et al., 2004; Susani et al., 2004; Hobson et al., 2006; Caux-Moncoutier et al., 2009; Olsen et al., 2014; Vemula et al., 2014; Peterlongo et al., 2015). Strengths of one or more splice sites may be altered and, in some instances, concomitant with amino acid changes in coding sequences (Rogan et al., 1998; Peterlongo et al., 2015). Information analysis has been a successful approach for recognizing nondeleterious, sometimes polymorphic variants (Rogan and Schneider, 1995; Colombo et al., 2013), and for distinguishing of milder from severe mutations (Rogan et al., 1998; von Kodolitsch et al., 1999; Lacroix et al., 2012).

Predicting the relative abundance of various transcripts by information analysis requires integration of the contributions of all pertinent cis-acting regulatory elements. We have applied quantitative methods to prioritize inferences as to which SNPs impact gene expression levels and transcript structure. Effects of mutations on combinations of splicing signals reveal changes in isoform structure and abundance (Mucaki et al., 2013; Caminsky et al., 2014). Multisite information theory-based models have also been used to detect and analyze SNP effects on cis-acting promoter modules that contribute to establishing transcript levels (Bi and Rogan, 2004; Vyhlidal et al., 2004; Lu et al., 2017; Lu and Rogan, 2019).

The robustness of this approach for predicting rare, deleterious splicing mutations justifies efforts to identify common SNPs that impact mRNA splicing. We previously described SNPs from dbSNP that affect splicing (Rogan et al., 1998; Nalla and Rogan, 2005). Here, we explicitly predict and validate SNPs that influence mRNA structure and levels of expression of the genes containing them in immortalized lymphoblastoid cell lines and tumors. Since constitutive splicing mutations can arise at other locations within pre-mRNA sequences that elicit cryptic splicing, we examined whether more common genomic polymorphisms might frequently affect the abundance and structure of splice isoforms.

## Methods

### Information Analysis

The protein-nucleic acid interactions intrinsic to splicing can be analyzed using information theory, which comprehensively and quantitatively models functional sequence variation based on a thermodynamic framework (Schneider, 1997). Donor and acceptor splice site strength can be predicted by the use of IT-based weight matrices derived from known functional sites (Rogan et al., 2003). The Automated Splice Site and Exon Definition server (ASSEDA) is an online resource based on the hg19 coordinate system to determine splice site information changes associated with genetic diseases (Mucaki et al., 2013). ASSEDA is now part of the MutationForecaster (http://www.mutationforecaster.com) variant interpretation system.

### Creation of Exon Array Database

Exon-level microarrays have been used to compare abnormal expression for different cellular states, which can then be confirmed by q-RT-PCR (Thorsen et al., 2008). We hypothesized that the predicted effect of SNPs on expression of the proximate exon would correspond to the expression of exon microarray probes of genotyped individuals in the HapMap cohort. We used the dose-dependent expression of the minor allele to qualify SNPs for subsequent information analysis consistent with alterations of mRNA splicing. Additional SNPs predicted by information analysis were also tested for effects on splicing (Nalla and Rogan, 2005).

Expression data were normalized using the PLIER (Probe Logarithmic Intensity Error) method on Affymetrix Human Exon 1.0 ST microarray data for 176 genotyped HapMap cell lines (Huang et al., 2007, Gene Expression Omnibus accession no. GSE 7792; Nembaware et al., 2008). Microarray probes which overlap SNPs, that were subsequently removed, were identified by intersecting dbSNP129 with probe coordinates [obtained from X:MAP (Yates et al., 2008) using the Galaxy Browser (Giardine et al., 2005)]. A MySQL database containing the PLIER normalized intensities and CEU (Utah residents with Northern and Western European ancestry) and YRI (Yoruba in Ibadan) genotypes for Phase I+II HapMap SNPs was created. Tables were derived to link SNPs to their nearest like-stranded probeset (to within 500 nt), and to associate probesets to the exons they may overlap (transcript and exon tables from Ensembl version 51). A MySQL query was used to create a table containing the splicing index (SI; intensity of a probeset divided by the overall gene intensity) of each probeset for each HapMap individual.

The database was queried to identify significant SI changes of an exonic probeset based on the genotype of a SNP the probeset was associated with (SNP within natural donor/acceptor region of exon). Probesets displaying a stepwise change in mean SI (where the mean SI of the heterozygous group is in between the mean SI values of the two homozygous groups) were identified using a different program script (criteria: the mean SI of homozygous rare and heterozygous groups are < 90% of the homozygous common group). Splicing Index boxplots were created with R, where the x- and y-axis are genotype and SI, respectively (**Supplementary Image 1**). These boxplots analyze the effect a SNP has on a particular probeset across all individuals.

SNPs with effects on splicing were validated by q-RT-PCR of lymphoblastoid cell lines. Where available, results were also compared to abnormal splicing patterns present in RNAseq data from tumors carrying these same SNPs (in the ValidSpliceMut database; Shirley et al., 2019). SNPs predicted to exhibit nominal effects on splicing (Δ*R_i_* < 1 bit) were included to determine minimal detectable changes by q-RT-PCR.

### Cell Culture and RNA Extraction

EBV-transformed lymphoblastoid cell lines of HapMap individuals with our SNPs of interest (homozygous common, heterozygous and homozygous rare when available) were ordered from the Coriell Cell Repositories (CEU: GM07000, GM07019, GM07022, GM07056, GM11992, GM11994, GM11995, GM12872; YRI: GM18855, GM18858, GM18859, GM18860, GM19092, GM19093, GM19094, GM19140, GM19159). Cells were grown in HyClone RPMI-1640 medium [15% FBS (HyClone), 1% L-Glutamine, and 1% Penicillin:streptomycin (Invitrogen); 37°C, 5% CO_2_]. RNA was extracted with Trizol LS (Invitrogen) from 10^6^ cells and treated with DNAase [20 mM MgCl_2_ (Invitrogen), 2 mM DTT (Sigma-Aldrich), 0.4 U/μL RNasin (Promega), 10 µg/ml DNase (Worthington Biochemical) in 1x TE buffer] at 37°C for 15 min. The reaction was stopped with EDTA (0.05 M; 2.5% v/v), and heated to 65°C for 20 min, followed by ethanol precipitation (resuspended in 0.1% v/v DEPC-treated 1x TE buffer). DNA was extracted using a Puregene Tissue Core Kit B (Qiagen).

### Design of Real-Time Expression Assays

Sequences were obtained from UCSC and Ensembl. DNA primers used to amplify a known splice form, or one predicted by information analysis, were designed using Primer Express (ABI). DNA primers (**Supplementary Table 1**) were obtained from IDT (Coralville, IA, USA), and dissolved to 200 uM. Primers were placed over junctions of interest to amplify a single splice form. T_m_ ranged from 58°C–65°C, and amplicon lengths varied from 69–136 nt. BLASTn (Refseq_RNA database) was used to reduce possible cross-hybridization. Primers were designed to amplify the wildtype splice form, exon skipping (if a natural site is weakened), and cryptic site splice forms which were either previously reported (UCSC mRNA and EST tracks) or predicted by information analysis (where *R_i_* cryptic site ≥ *R_i_* weakened natural site).

Two types of reference amplicons were used to quantify allele specific splice forms. These consisted of intrinsic products derived from constitutively spliced exons with the same gene and external genes with high uniformity of expression among HapMap cell lines. Reference primers internal to the genes of interest were designed 1–4 exons adjacent from the affected exon (exons without any evidence of variation in the UCSC Genome Browser; Kent et al., 2002), placed upstream of the SNP of interest whenever possible. Two advantages to including an internal reference in the q-RT-PCR experiment include: potential detection of changes in total mRNA levels; and account for inter-individual variation of expression.

External reference genes (excluding the SNP of interest) were chosen based on consistent PLIER intensities with low coefficients of variation in expression among all 176 HapMap individuals. The following external controls were selected: exon 39 of *SI* (PLIER intensity 11.4 ± 1.7), exon 9 of *FRMPD1* (22 ± 2.81), exon 46 of *DNAH1* (78.5 ± 9.54), exon 3 of *CCDC137* (224 ± 25), and exon 25 of *VPS39* (497 ± 76). The external reference chosen for an experiment was matched to the intensity of the probeset within the exon of interest. This decreased potential errors in ΔΔC_T_ values and proved to be accurate and reproducible for most genes.

To control for interindividual variation in expression, we compared expression in HapMap individuals based on their SNP genotypes and familial relatedness. Families with all three possible genotypes were available (homozygous common, rare, and heterozygous) for 12 of these SNPs (rs1805377, rs2243187, rs2070573, rs2835655, rs2835585, rs2072049, rs1893592, rs6003906, rs1018448, rs13076750, rs16802, and rs8130564). For those families in which all genotypes were not represented, samples from the same ethnic background (YRI or CEU populations) were compared for the missing genotype (N = 8; rs17002806, rs2266988, rs1333973, rs743920, rs2285141, rs2838010, rs10190751, rs16994182; individuals with homozygous common and rare genotypes were from the same families for the latter two SNPs). Two SNPs were tested using homozygous individuals from different ethnic backgrounds: rs3747107 (*GUSBP11*) and rs2252576 (*BACE2*). While the splicing impact of rs3747107 was clearly observable by q-RT-PCR, either background or data noise did impact the interpretation of effects of rs2252576.

### PCR and Quantitative RT-PCR

M-MLV reverse transcriptase (Invitrogen) converted 1µg of DNase-treated RNA to cDNA with 20 nt Oligo-dT (25µg/ml; IDT) and rRNAsin (Promega). Precipitated cDNA was resuspended in water at 20 ng/µl of original RNA concentration. All designed primer sets were tested with conventional PCR to ensure a single product at the expected size. PCR reactions were prepared with 1.0 M Betaine (Sigma-Aldrich), and were heated to 80°C before adding Taq Polymerase (Invitrogen). Optimal T_m_ for each primer set was determined to obtain maximum yield.

Quantitative PCR was performed with an Eppendorf Mastercycler ep Realplex 4, a Bio-Rad CFX96, as well as a Stratagene Mx3005P. SYBR Green assays were performed using the KAPA SYBR FAST qPCR kit (Kapa Biosystems) in 10 µl reactions using 200 µM of each primer and 24 ng total of cDNA per reaction. For some tests, SsoFast EvaGreen supermix (Bio-Rad) was used with 500 µM of each primer instead.

When testing the effect of a SNP, amplification reactions with all primers designed to detect all relevant isoforms (as well as the gene internal reference and external reference) were run simultaneously, in triplicate. C_t_ values obtained from these experiments were normalized against the same external reference using the Relative Expression Software Tool (REST; http://www.gene-quantification.de/rest.html; Pfaffl et al., 2002).

### Taqman Assay

Two dual-labeled Taqman probes were designed to detect the two splice forms of *XRCC4* (detecting alternative forms of exon 8 either with or without a 6 nt deletion at the 5' end). Probes were placed over the sequence junction of interest where variation would be near the probe middle (**Supplementary Table 1**). The assay was performed on an ABI StepOne Real-Time PCR system using ABI Genotyping Master Mix. Experiment was run in 25 µl reactions (300 nM each primer, 400nM probe [5'-FAM or TET fluorophore with a 3' Black Hole quencher; IDT], and 80 ng cDNA total). Probes were tested in separate reactions.

### RNAseq Analyses

The previous analyses were extended to include 24 additional, common SNPs for their potential influence on splicing. All SNVs present in ICGC (International Cancer Genome Consortium) patients (Shirley et al., 2019) were evaluated by the Shannon Pipeline (SP; Shirley et al., 2013) to identify those altering splice site strength. Common SNPs (average heterozygosity > 10% in dbSNP 150) predicted to decrease natural splice site strength by SP (where *ΔR_i_* < −1 bit) were selected. ICGC patients carrying these flagged SNPs were identified, and the expression of the corresponding SNP-containing region in RNAseq was visualized with IGV (Integrated Genome Viewer; https://igv.org; Robinson et al., 2017). Similar RNAseq reads were grouped using IGV collapse and sort commands, which caused nonconstitutive spliced reads to cosegregate to the top of the viewing window. IGV images which did not meet our gene expression criteria (exon affected by the SNP must have ≥5 RNAseq reads present) were eliminated. As this generated thousands of images, we report the analysis of two ICGC patients [DO47132 (Renal Cell Cancer) and DO52711 (Chronic Lymphocytic Leukemia)], chosen randomly, preselecting tissues to increase the likelihood of finding expression in these regions. Images were evaluated sequentially (in order of rsID value) and only concluded once the first 24 SNPs meeting these criteria were found. This type of analysis could not reveal a splicing event to be more abundant in these patients when compared to noncarriers. Nevertheless, splicing information changes resulting from SNPs corresponded to observed alternative and/or other novel splice isoforms. We then queried the ValidSpliceMut database for these SNPs, as abnormal splicing was only flagged in the database when the junction-read or read-abundance counts significantly exceeded corresponding evidence type in a large set of normal control samples (Shirley et al., 2019).

## Results

### Selection of Candidate SNPs Affecting Splicing

A publicly available exon microarray dataset was initially used to locate exons affected by SNPs altering splice site strength. A change in the mean SI of a diagnostic probeset in individuals of differing genotypes at the same variant can suggest altered splicing. The increase or decrease in SI is related to the expected impact of the SNP on splicing. For example, an exonic probe which detects a normally spliced mRNA will have decreased SI in the event of skipping. Mean SI may be increased when a probe detects the use of an intronic cryptic splice site. SNPs with strong impact on splicing will distinguish mean SI levels of individuals homozygous for the major versus minor alleles (and with heterozygous genotypes).

There were 9,328 HapMap-annotated SNPs within donor/acceptor regions of known exons which contained at least one probeset. Of 987 SNPs that are associated to exonic probesets which differ in mean SI between the homozygous common and rare HapMap individuals, 573 caused a decrease in natural site *R_i_* value. Inactivating and leaky splicing variants (reduction in information content where final *R_i_* ≥ *R_i,minimum_* [minimum functional splice site strength]) both exhibit reduced SI values and were similarly abundant. Thus, both severe and moderate splicing mutations with reduced penetrance and milder molecular phenotypes were detected, consistent with Mendelian disorders (von Kodolitsch et al., 1999; von Kodolitsch et al., 2006).

Of the SNPs associated with significant changes in *R_i_* (termed Δ*R_i_*), 9,328 occurred within the natural splice sites of exons detectable with microarray probesets. We initially focused on 21 SNPs on chromosome 21 (0.23% total, 18.8% of chr21) and 34 on chromosome 22 (0.36% of total, 14.5% of chr22) associated with stepwise decreases in probeset intensity at each genotype. Seven of the chr21 SNPs and nine of the chr22 SNPs caused information changes with either natural splice site Δ*R_i_* ≥ 0.1 bits, or cryptic site(s) with an *R_i_* value comparable to a neighbouring natural site, and in which mRNA or EST data supported use of the cryptic site. These SNPs included: rs2075276 [*MGC16703*], rs2838010 [*FAM3B*], rs3747107 [*GUSBP11*], rs2070573 [*C21orf2*], rs17002806 [*WBP2NL*], rs3950176 [*EMID1*], rs1018448 [*ARFGAP3*], rs6003906 [*DERL3*], rs2266988 [*PRAME*], rs2072049 [*PRAME*], rs2285141 [*CYB5R3*], rs2252576 [*BACE2*], rs16802 [*BCR*], rs17357592 [*COL6A2*], rs16994182 [*CLDN14*], and rs8130564 [*TMPRSS3*].

**Figure 1:**
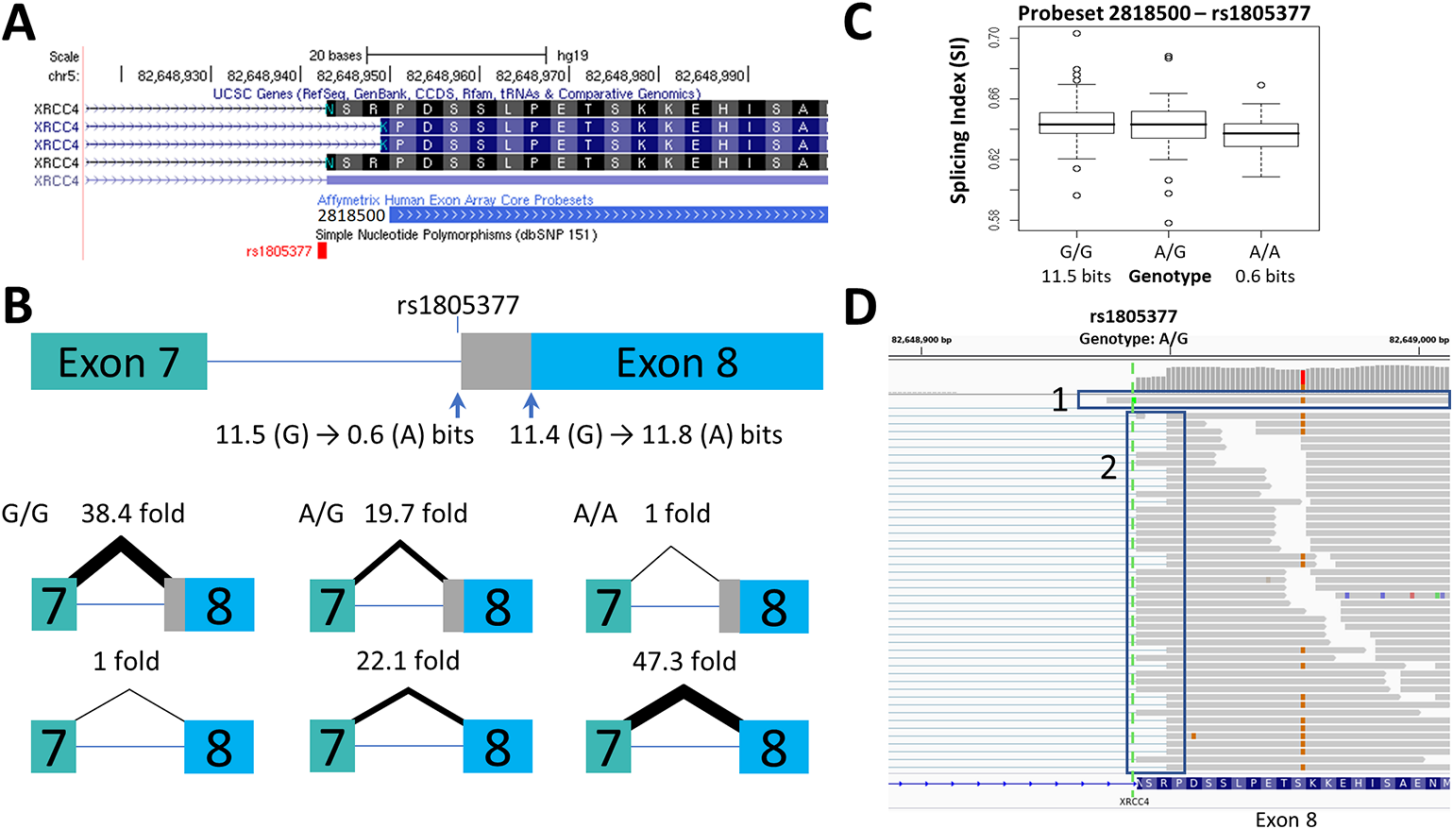
Splicing Impact of rs1805377 (*XRCC4*). The natural acceptor of *XRCC4* exon 8 is abolished by rs1805377 (11.5 −> 0.6 bits) while simultaneously strengthening a second exonic cryptic acceptor 6nt downstream (11.4 to 11.8 bits), resulting in a 6nt deletion in the mRNA. **(A)** Both of these acceptor sites have been validated in GenBank mRNAs, i.e., NM_022406 and NM_003401 (UCSC panel derived from http://genome.ucsc.edu). **(B)** The relative abundance of the two splice forms was determined by q-RT-PCR. The weaker rs1805377 A/A genotype (0.6 bit acceptor) was used ~47-fold less frequently than the cryptic downstream acceptor (11.8 bits). **(C)** The two splice isoforms cannot be distinguished by the exon microarray as the upstream probeset (ID 2818500) does not overlap the variable region, though the average expression of the rs1805377 A/A genotype is reduced. **(D)** ValidSpliceMut flagged this mutation for intron retention, which can be observed in the RNAseq of heterozygous ICGC patient DO27779 [Box 1]. Use of both acceptor sites is also evident [Box 2]. For more detail for this and all of the other single nucleotide polymorphisms (SNPs) analyzed, refer to **Supplementary Image 1**.

**Figure 2:**
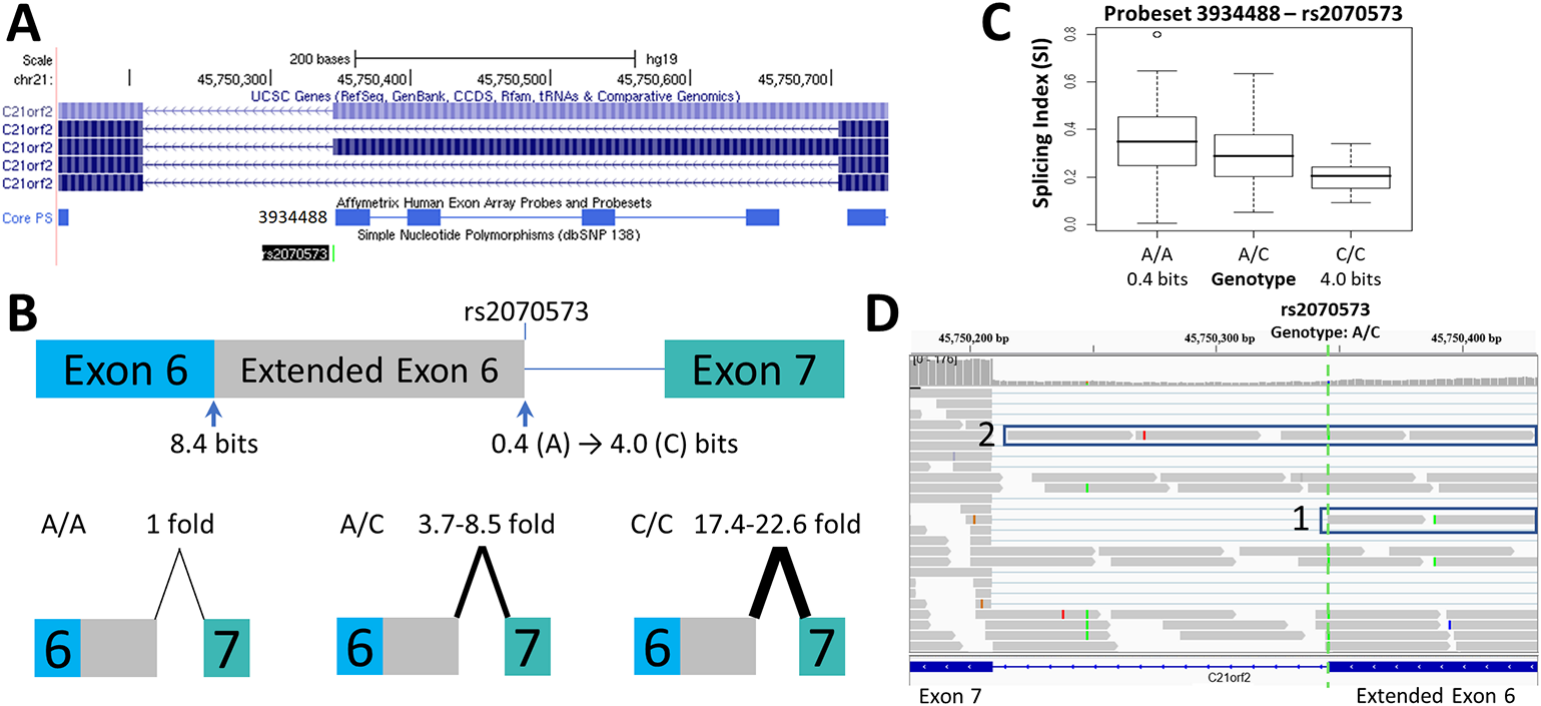
Splicing Impact of rs2070573 (*C21orf2*). **(A)** The single nucleotide polymorphisms (SNP) rs2070573 is a common polymorphism which alters the first nucleotide of the extended form of *C21orf2* exon 6. **(B)** The donor site is strengthened by the presence of the C-allele (*R_i_* 0.4 to 4.0 bits; A > C) and its use extends the exon by 360 nt. Q-RT-PCR found a ~4-9-fold and ~17-23-fold increase in the extended exon 6 splice form in the A/C and C/C cell lines tested, respectively. **(C)** The exon microarray probeset which detects the extension (ID 3934488) shows a stepwise increase in SI with C-allele individuals which supports the q-RT-PCR result**. (D)** The variant was present in ValidSpliceMut, which associated the A-allele with an increase in total intron retention [six patients flagged for total intron retention read abundance; p=0.019 (average over all patients)]. This image displays sequence read distributions in the RNAseq data of TCGA BRCA patient, TCGA-BH-A0H0, who is heterozygous for rs2070573. The IGV panel indicates reads corresponding to total intron 6 retention [Box 1] and which extend beyond the constitutive donor splice site of exon 6 into the adjacent intron [Box 2]. All 4 reads which extend over the exon splice junction are derived from the G-allele (strong binding site; not all visible in panel **D**).

**Figure 3:**
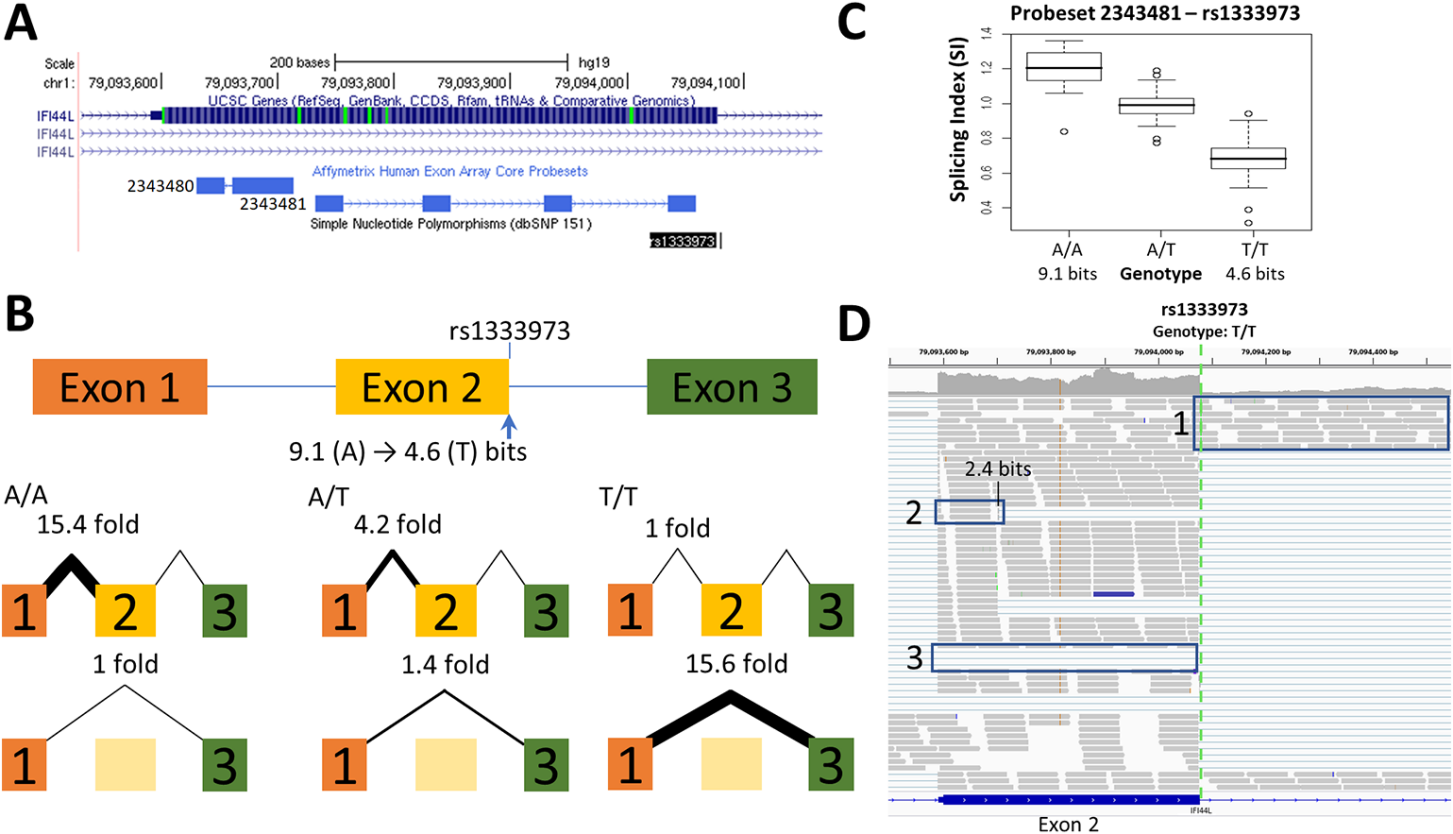
Splicing Impact of rs1333973 (*IFI44L*). **(A)** The natural donor of *IFI44L* exon 2 is weakened from 9.1 to 4.6 bits (A > T) by rs1333973, increasing the frequency of exon 2 skipping and other events. **(B)** By q-RT-PCR, skipping was found to be 15.6-fold higher while normal splicing was 15.4-fold lower in A homozygotes (relative to T homozygotes). **(C)** Exon microarray data strongly supports the q-RT-PCR findings. **(D)** ICGC patient DO6354 is homozygous for the T-allele, which resulted in exon 2 skipping [Box 3], failure to recognize the exon 2 donor causing total retention of intron 2 [Box 1], and activation of an upstream exonic 2.4 bit cryptic donor 375 nt from the affected site [Box 2].

The minimum information change for detecting a splicing effect by expression microarray is constrained by several factors. Detection of splice isoforms can be limited by genomic probeset coverage, which cannot distinguish alternative splicing events in close proximity (see **Figure 1A**). Even where genotype-directed SI changes are very distinct, some individuals with the common allele have equivalent SI values to individuals with the rare allele [rs2070573 (**Figure 2**) and rs1333973 (**Figure 3**)]. In some cases, the number of individuals with a particular genotype is insufficient for statistical significance (rs2243187; **Supplementary Image 1.4**). Although exon microarrays can be used to find potential alternate splicing and give support to our predictions, it became necessary to validate the microarray predictions by q-RT-PCR, TaqMan assays, and with RNAseq data from SNP carriers.

We report q-RT-PCR validation studies for 13 of the 16 SNPs (q-RT-PCR primers could not be designed for rs16994182, rs2075276, and rs3950176), along with nine other candidate SNPs from our previous information theory-based analyses (Nalla and Rogan, 2005): rs1805377 [*XRCC4*], rs2243187 [*IL19*], rs2835585 [*TTC3*], rs2865655 [*TTC3*], rs1893592 [*UBASH3A*], rs743920 [*EMID1*], rs13076750 [*LPP*], rs1333973 [*IFI44L*], and rs10190751 [*CFLAR*].

After amplification of known and predicted splice forms (**Supplementary Table 1**), 15 SNPs showed measurable changes in splicing consistent with information-theory predictions. Ten increase alternate splice site use (two of which increased strength of cryptic site, eight activated an unaffected pre-existing cryptic site), six affect exon inclusion (five increased exon skipping), three increased activation of an alternative exon, and four decreased overall expression levels. Altered splicing could not be validated for six SNPs, however experimental analyses of three of the five SNPs where Δ*R_i_* < 1 bit were hampered by high interindividual variability in expression.

Changes in splice site information were used to predict observed differences in splice isoform levels (**Table 1**). **Figures 1**–**3** and **Supplementary Image 1** indicate the experimentally-determined splicing effects for each SNP, a modified UCSC Genome Browser image of the relevant region, boxplots showing exon microarray expression levels of each allele for the relevant probesets, and an IGV image of the RNAseq results for an individual tumor carrying the SNP. Abundance of the aberrant splice forms measured by q-RT-PCR (relative to an internal gene reference) is indicated in **Table 2**. Changes in predicted splice site strength were consistent with results measured by q-RT-PCR for 12 out of the 15 SNP (exceptions were rs2070573, rs17002806, and rs2835585). Variants predicted to reduce strength ≥ 100-fold were found to reduce expression by 38- to 58-fold, the variance falling within the margin of measurement error. Modest natural splice site affinity changes predicted to be < eightfold (ΔR_i_ < 3.0) did not consistently result in detectable changes in splicing. In some instances, lower abundance splice forms were observed (i.e. rs2835585 altered exon skipping levels by up to 8.8-fold; nevertheless, the normal splice form predominated).

**Table 1:** Summary of q-RT-PCR results.

Summary of q-RT-PCR results					SNP effects (Increase/Decrease in fold change of homozygotes)						Additional Expression Evidence	
Gene	Supplementary Image 1 panel / rsID / HGVS notation	Splice type	Information Change (n–natural, c–cryptic site)	Fold change	Natural site	Cryptic site	Exon skipping	Alternate exon	Total mRNA	In-frame^a^	Exon microarray	RNAseq (Valid-SpliceMut)
*XRCC4*	S1.6/ rs1805377 / NM_003401: c.894-7G>A NM_022406: c.894-1G>A	A	11.5 (G) -> 0.6 (A) (n)	1886	38.4	–	–	–	n/a^b^	Y	N	Y
		A	11.4 (G) -> 11.8 (A) (n)	1.3	–	47.3^c^	–	–	n/a^b^	Y	N	Y
*IL19*	S1.4/ rs2243187 / NM_013371: c.364-1G>A	A	4.7 (G) -> -6.2 (A) (n)	26.3	1.8^d^	1.8^d^	2.1^d^	–	n/a^b^	Y	Y	N
*C21orf2*	S1.1/ rs2070573 / NM_004928: c.643-137A>C	D	0.4 (A) -> 4.0 (C) (c)	12.1	NC	22.6	–	–	n/a^b^	Y	Y	Y
*TTC3*	S1.21/ rs2835655 / NM_003316: c.5115G>A	D	11.2 (G) -> 8.2 (A) (n)	8.1	1.5	–	1.3^e^	–	1.5	–	N	N
*TTC3^f^*	S1.5/ rs2835585 / NM_003316: c.188-8T>A	A	7.6 (T) -> 5.4 (A) (n)	4.5	NC	–	8.8	–	n/a^b^	Y	N	Y
*WBP2NL*	S1.13/ rs17002806 / NM_152613: c.*86G>A	D	9.2 (G) -> 6.0 (A) (n)	9.1	23.8	34+^g^	–	–	n/a^b^	N	Y	N
*GUSBP11*	S1.8/ rs3747107 / NR_024448: n.2961+6512 C>G	A	4.7 (C) -> -7.0 (G) (n)	25.9	98.3	31/42.8^h^	–	1.6	n/a^b^	Y/N^i^	N	N
*PRAME*^j^	S1.9/ rs2266988^k^ / NM_006115: c.19G>A	D	7.8 (G) -> 6.2 (A) (n)	3.0	NC	–	8.8	–	n/a^b^	N	N	N
*PRAME*^j^	S1.14/ rs2072049 / NM_006115: c.954-16G>T	A	8.2 (G) -> 7.1 (T) (n)	2.2	2.6^d^	–	–	–	3.1^d^	-	Y	Y
*UBASH3A*	S1.10/ rs1893592 / NM_018961: c.1393+3A>C	D	8.7 (A) -> 4.2 (C) (n)	22.9	3.0	2.0/1.7^l^	N/A^m^	–	1.4	Y	N	Y
*DERL3*	S1.15/ rs6003906 / NM_198440: c.328-8A>T	A	–2.1 (A) -> -4.3 (T) (n)	4.6	NC	2.0	–	–	n/a^b^	N	Y	N
*ARFGAP3*	S1.3/ rs1018448 / NM_014570: c.1065C>A	A	10.5 (C) -> 8.3 (A) (n)	4.5	2.2	–	1.4	–	2.0	Y	Y	N
*CFLAR*	S1.2/ rs10190751 / NM_003879: c.606+934G>A NM_001127184:c.607-1G>A	A	16.1 (G) -> 5.2 (A) (n)	1885	10000+	–	–	2.1	n/a^b^	Y	Y	Y
*IFI44L*	S1.11/ rs1333973 / NM_006820: c.478+3A>T	D	9.1 (A) -> 4.6 (T) (n)	22.6	15.4	–	15.6	–	3.9	N	Y	N
*LPP*	S1.12/ rs13076750 / NM_001167671:c.-66-8G>A	A	9.3 (G) -> -1.6 (A) (n)	625	5000+	15.8	26.6	–	–	Y	N	Y
*EMID1*	S1.7/ rs743920 / NM_133455: c.326C>G	A	6.0 (C) -> 7.9 (G) (c)	3.6	n/a^b^	5.8	–	–	n/a^b^	–	Y	N
*CLDN14*	S1.22/ rs16994182 / NM_001146078:c.-82+4C>G	D	7.4 (C) -> 6.8 (G) (n)	1.6	5.9	–	2.1	–	n/a^b^	Y	N/A	N
*BCR*	S1.17/ rs16802 / NM_004327:c.2708-13A>G	A	5.6 (A) -> 5.8 (G) (n)	1.1	NC^n^	–	–	–	n/a^b^	–	N	N
*TMPRSS3*	S1.19 / rs8130564 / NM_032404: c.66-13T>C	A	4.2 (T) -> 4.4 (C) (n)	1.1	NC^n^	–	–	–	n/a^b^	–	N	N
*BACE2*	S1.18/ rs2252576 / NM_012105: c.748-10C>T	A	7.2 (C) -> 8.0 (T) (n)	1.5	NC^n^	–	–	–	n/a^b^	–	N	N
*CYB5R3*	S1.20/ rs2285141 / NM_007326: c.-48-18G>T	A	2.0 (G) -> 1.2 (T) (n)	1.7	NC	–	–	1.8	n/a^b^	–	N	N
*FAM3B*	S1.16/ rs2838010 / NM_058186: c.20-217A>T	D	–10.8 (A) -> 7.8 (T) (c)	228	–	–	–	N/A^i^	n/a^b^	Y	N	N

Red text indicates a decrease in the abundance of a particular splice form, while green text indicates an increase in abundance. A – Acceptor Splice Site Affected; D – Donor Splice Site Affected; NC - Not detectable (abolished). ^a^ Splicing events which alter reading frame may induce nonsense-mediated decay; ^b^ No allele specific difference in expression and splicing; ^c^ complete discrimination of both isoforms using a custom designed TaqMan probe; ^d^ Values from comparing heterozygote with homozygote common; ^e^ Change in splicing likely related to change in RNA level; ^f^ Intron 2-3 retention of *TTC3* amplified by PCR, but no allele specific change detected; ^g^ This splice form not at detectable levels in homozygote; ^h^ Cryptic acceptor 114nt upstream of affected site / cryptic acceptor 118nt upstream of affected site; ^i^ mRNA in-frame when alternate exon is used, and out of frame due to cryptic site use; ^j^ *PRAME* is a special case where two SNPs affect splicing of two separate exons; ^k^ rs2266988 and rs1129172 are identical SNPs on opposite strands; ^l^ Cryptic donor 555nt downstream of affected site / cryptic donor 29nt downstream of affected site; ^m^ Splice form not detected by PCR; ^n^ High variability between individuals with the same genotype by q-RT-PCR.

**Table 2:** Abundance of mRNA splice forms relative to internal gene reference.

Gene	rsID	mRNA Splice Form	Homozygotes strong allele (%) [# Patients Tested]	Homozygotes weak allele (%) [# Patients Tested]
*C21orf2*	rs2070573	Extended Exon 6 Splice Form	45.3 ± 16.9 [2]^1^	1.5 ± 0.2 [2]
*TTC3*	rs2835585	Exon 3 Skipping	<0.1 ± 0.0 [2]	0.3 ± 0.1 [2]
*IL19^2^*	rs2243187	3 nt Inclusion of Exon 5	~100 [1]	53.2 ± 3.0 [1; Het.]
*IL19^2^*	rs2243187	3 nt Exclusion of Exon 5	61.0 ± 3.4 [1]	~100 [1; Het.]
*IL19^2^*	rs2243187	Exon 5 Skipping	14.1 ± 1.4 [1]	6.8 ± 1.1 [1; Het.]
*UBASH3A*	rs1893592	29 nt Retention of Intron 10	8.6 ± 4.6 [1]	4.2 ± 5.8 [1]
*XRCC4*	rs1805377	6 nt Inclusion of Exon 8	~100 [1]	3.1 ± 0.5 [1]
*XRCC4*	rs1805377	6 nt Exclusion of Exon 8	2.6 ± 1.1 [1]	61.4 ± 26.3 [1]
*PRAME*	rs2266988	Normal Exon 3 Splicing	32.4 ± 3.6 [1]	20.1 ± 3.9 [1]
*PRAME*	rs2266988	Exon 3 Skipping	1.2 ± 1.2 [1]	3.5 ± 3.0 [1]
*GUSBP11*	rs3747107	Exon 8 Splicing	34.0 ± 0.6 [1]	0.3 ± 0.0 [2]
*GUSBP11*	rs3747107	Alternative Exon 8	37.3 ± 7.3 [1]	39.8 ± 3.7 [2]
*GUSBP11*	rs3747107	114 nt Retention of Intron 7	0.1 ± 0.0 [1]	1.4 ± 0.1 [2]
*GUSBP11*	rs3747107	118 nt Retention of Intron 7	0.2 ± 0.0 [1]	8.0 ± 0.5 [2]
*DERL3*	rs6003906	Normal Exon 5 Splicing	~100 [1]	55.8 ± 0.0 [1]
*DERL3*	rs6003906	Extended Exon 4; Short Exon 5	3.2 ± 0.0 [1]	4.0 ± 0.0 [1]
*ARFGAP3*	rs1018448	Exon 12 Skipping	12.3 ± 5.7 [3]	23.1 ± 10.1 [1]
*IFI44L*	rs1333973	Normal Exon 2 Splicing	57.0 ± 0.0 [1]	14.7 ± 0.0 [1]
*IFI44L*	rs1333973	Exon 2 Skipping	0.8 ± 0.0 [1]	48.0 ± 0.0 [1]
*CFLAR*	rs10190751	Upstream Exon 7 Use	~100 [1]	<0.1 ± 0.0 [1]
*CFLAR*	rs10190751	Downstream Exon 7 Use	39.0 ± 0.0 [1]	87.1 ± 0.0 [1]
*WBP2NL*	rs17002806	25 nt Intron 6 Retention	N.D.^2^	2.3 ± 0.0 [1]
*CYB5R3*	rs2285141	Alternate Exon 2 Use	<0.1 ± 0.0 [1]	<0.1 ± 0.0 [1]
*EMID1*	rs743920	6 nt deletion of Exon 4	69.0 ± 9.7 [1]	9.2 ± 1.4 [1]
*CLDN14*	rs16994182	Exon 2 Skipping	1.9 ± 0.0 [1]	5.3 ± 0.0 [1]

^1^Average expression was computed by comparing qPCR C_t_ values across multiple experimental runs and normalized against C_t_ of internal gene reference. SNPs tested in multiple experiments with one individual of each genotype will have a standard deviation of 0.0. ^2^Heterozygote; Individuals who are homozygous for IL19 SNP rs2243187 were not available for testing. N.D., Not detected. C_t_ values were not available for LPP rs13076750.

### SNPs Affecting Cryptic Site Strength and Activity

Increased cryptic site use coinciding with a decrease in natural site strength (**Table 1**) was validated for: rs1805377 (**Figure 1**); rs2243187 (**Supplementary Image 1.4**); rs3747107 (**Supplementary Image 1.8**); rs17002806 (**Supplementary Image 1.13**); rs6003906 (**Supplementary Image 1.15**); and rs13076750 (**Supplementary Image 1.12**). rs2070573 (**Figure 2**) and rs743920 (**Supplementary Image 1.7**) strengthened cryptic splice sites resulting in increased use of these sites. Despite the difference in strength between the natural and cryptic sites affected by rs743920, the upstream 2.4 bit site was used more frequently (**Table 2**). Both *IL19* and *XRCC4* regions tested showed preference to the upstream acceptor as well, which is consistent with the processive mechanism documented to recognize acceptor splice sites (Robberson et al., 1990).

### SNPs Affecting Exon Inclusion

SNPs that reduced natural site strength (Δ*R_i_* from 1.6 to 10.9 bits) increased exon skipping from 2- to 27-fold for homozygotes of differing genotypes of rs2835585 (**Supplementary Image 1.21**), rs1018448 (**Supplementary Image 1.3**), rs1333973 (**Figure 3**), rs2266988 (**Supplementary Image 1.9**), and rs13076750 (**Supplementary Image 1.12**). The exon microarray probesets for rs1018448 and rs1333973 detect decreased expression by genotype, which is consistent with increased exon skipping. Changes of average SI values did not correspond as well to specific genotypes for rs2835585 (*TTC3)*, rs2266988 (*PRAME)*, and rs13076750 (*LPP)*, possibly due to increased cryptic site use (*LPP*) or large differences in the abundance of constitutive and skipped isoforms (*PRAME*, *TTC3*).

### SNPs Promoting Alternate Exon Use

SNP-related decreases in natural splice site strength may promote the use of alternative exons up or downstream of the affected exon. rs10190751 (**Supplementary Image 1.2**) is known to modulate the presence of the shorter c-FLIP(S) splice form of *CFLAR* (Ueffing et al., 2009). The use of this exon differed by 2^17^-fold between the strong and weak homozygotes tested, which was reflected by the expression microarray result. By q-RT-PCR, the *CFLAR* (L) form using an alternate downstream exon was found to be 2.1-fold more abundant in the homozygote with the weaker splice site. rs3747107 (**Supplementary Image 1.8**) and rs2285141 (**Supplementary Image 1.20**) exhibit evidence of an increased preference by q-RT-PCR for activation of an alternate exon, though the microarray results for the corresponding genotypes for both SNPs were not significantly different.

### SNP-Directed Effects on mRNA Levels

A change in the strength of a natural site of an exon can affect the quantity of the processed mRNA (Caminsky et al., 2014). This decrease in mRNA could be caused by nonsense mediated decay (NMD), which degrades aberrant transcripts that would result in premature protein truncation (Cartegni et al., 2002). Of the 22 SNPs tested, 2 showed a direct correlation between a decrease in natural splice site strength, reduced amplification of the internal reference by q-RT-PCR (of multiple individuals) and a decreasing trend in expression by genotype by microarray: rs2072049 (**Supplementary Image 1.9**) and rs1018448 (**Supplementary Image 1.3**), although these differences do not meet statistical significance.

### SNPs With Pertinent Splicing Effects Detected by RNAseq

Evidence for impact on splicing of the previously described SNPs was also assessed in TCGA and ICGC tumors by high throughput expression analyses. Splicing effects of these variants detected by q-RT-PCR and RNAseq were concordant in 80% of cases (N = 16 of 20 SNPs), while impacts of 10% of SNPs (N = 2) were partially concordant as a result of inconsistent activation of cryptic splice sites (**Supplementary Images 1.8D** and **1.10D**). Several isoforms predicted by information analysis of these SNPs were present in complete transcriptomes, but were undetectable by q-RT-PCR or expression microarrays. Examples include a 4.9 bit cryptic site activated by rs3747107 located 2 nucleotides from the natural splice site (**Supplementary Image 1.8D**), exon skipping by rs1893592 (**Supplementary Image 1.10D**), and a cryptic exon activated by rs2838010 (**Supplementary Image 1.16C**). Processed mRNAs that were not detected by q-RT-PCR may have arisen as a result of a lack of sensitivity of the assay, to NMD (which could mask detection of mis-splicing), to a deficiency of an undefined trans-acting splicing factor, or to design limitations in the experimental design. Another possibility is that the discordant splicing patterns of these two SNPs based could potentially be related to differences in tissue origin, since only the RNAseq findings were tumor-derived, whereas results obtained by the other approaches were generated from RNA extracted from lymphoblastoid cell lines. Cell culture conditions such as cell density and phosphorylation status can affect alternative splicing patterns (Li et al., 2006; Szafranski et al., 2014). These conditions, however, have not been studied in cases of allele-specific, sequence differences at splice sites or cis-acting regulatory sites that impact splice site selection. Considering the high level of concordance of splicing effects for the same SNPs in uncultured and cultured cells, it seems unlikely that culture conditions significantly impacts the majority of allele-specific, alternatively spliced isoforms. Our information theory-based analyses show that the dominant effect of SNP genotypes is to dictate common changes in splice site strength regardless of cell origin.

The results obtained from q-RT-PCR and RNAseq data for rs2070573, rs10190751, rs13076750, rs2072049, rs2835585, rs1893592, and rs1805377 were complementary to findings based on RNAseq (**Supplementary Table 2**). RNAseq data can reveal potential allele-specific alternate splicing events that were not considered at the primer design phase of the study, while q-RT-PCR is more sensitive and can reveal less abundant alternative splice forms. A weak 0.4 bit splice site associated with rs2070573 was less abundant than the extended isoform (**Figure 2**) by both q-RT-PCR and exon microarray, however ValidSpliceMut also revealed increased total *C21orf2* intron 6 retention in five tumors with this allele. Similarly, rs10190751 was flagged for intron retention in 29 tumors, which was not evident by the other approaches. The long form of this transcript (c-FLIP[L]) in homozygous carriers of this SNP was twice as abundant by q-RT-PCR than the shorter allele, associated with the weak splice site. rs13076750 activates an alternate acceptor site for a rare exon that extends the original exon length by seven nucleotides. The exon boundary can also extend into an adjacent exon, based on RNAseq of eight tumors carrying this SNP. Expression was decreased in the presence of a 6.2 bit splice site derived from a rs2072049 allele that weakens the natural acceptor site of the terminal exon of *PRAME*. The actual cause of diminished expression is likely to have been related to NMD from intron retention. ValidSpliceMut showed intron retention to be increased in rs2835585, whereas increased exon skipping for the allele with the weaker splice site was demonstrated by q-RT-PCR. rs1893592 caused significant intron retention in all tumors (N = 9), with exon skipping present in 3 diffuse large B-cell lymphoma patients, which was not detected by q-RT-PCR. Finally, rs1805377 was associated with the significant abundance of read sequences indicating *XRCC4* intron 7 retention by RNAseq (N = 32), however this isoform could not be distinguished by the primers designed for q-RT-PCR and by TaqMan assay.

Alternative splicing events detected by RNAseq that were not evident in either q-RT-PCR or microarray studies included exon skipping induced by rs743920 (**Supplementary Image 1.7D**), activation of a preexisting cryptic splice site by rs1333973 (**Figure 3**), and intron retention by rs6003906 (**Supplementary Image 1.15D**). rs743920 creates an exonic hnRNP A1 site (*R_i_* = 2.8 bits) distant from the natural site which may compromise exon definition (Mucaki et al., 2013; Peterlongo et al., 2015) and may explain the SNP-associated increase in exon skipping. Exon definition analyses of total exon information (*R_i,total_*) also predicted the cryptic isoform arising from rs1333973 to be the most abundant (*R_i,total_* = 9.4 bits).

### Allele-Specific mRNA Splicing for Other SNPs Identified Through RNAseq

A distinct set of 24 high population frequency SNPs were also evaluated for their potential impact on mRNA splicing by RNAseq analysis of ICGC patients. Those resulting in significantly decreased natural splice site strength (Δ*R_i_* < −1 bit) were analyzed for SNP-derived alternative splicing events. SNPs fulfilling these criteria expressed at sufficient levels over the region of interest were: rs6467, rs36135, rs154290, rs166062, rs171632, rs232790, rs246391, rs324137, rs324726, rs448580, rs469074, rs518928, rs624105, rs653667, rs694180, rs722442, rs748767, rs751128, rs751552, rs752262, rs832567, rs909958, rs933208, and rs1018342 (**Table 3**). Splicing was predicted to be leaky for all natural splice sites affected by these SNPs (Rogan et al., 1998; *R_i,final_* ≥ 1.6 bits), where reduction in *R_i_* values ranged from 1.1 to 3.3 bits.

**Table 3:** RNAseq analysis of natural splice sites weakened by common single nucleotide polymorphisms (SNPs).

Gene	rsID^1^	HGVS Notation (HG19)^2^	*R_i_ initial*	*R_i_ final*	*ΔR_i_*	Alternative Splicing Observed
*CYP21A2*	rs6467	6:32006858C > A	6.1	4.5	−1.6	Intron Retention; Cryptic Site Use
*TRIM23*	rs36135	5:64890479A > C	10.0	7.5	−2.5	Intron Retention
*ZFYVE16*	rs166062	5:79773028T > G	14.1	11.8	−2.4	Intron Retention
*APBB3*	rs171632	5:139941318A > G	2.5	1.4	−1.1	Intron Retention; Cryptic Site Use
*SMIM8*	rs448580	6:88040399T > G	6.9	4.4	−2.5	Exon Skipping
*FCHSD1*	rs469074	5:141024136T > G	10.4	7.1	−3.3	Intron Retention
*KIFAP3*	rs518928	1:169890933A > G	15.6	14.5	−1.1	Intron Retention
*CEPT1*	rs694180	1:111726213A > G	9.6	7.0	−2.6	Intron Retention
*ADCY10P1*	rs722442	6:41089681A > G	7.5	4.9	−2.5	Exon Skipping
*MICAL1*	rs752262	6:109770999G > C	4.0	2.7	−1.4	Intron Retention
*MAP3K1*	rs832567	5:56152416C > A	6.8	5.0	−1.7	Intron Retention; Exon Skipping
*METTL13*	rs909958	1:171763522C > A	11.4	9.9	−1.4	Intron Retention
*DDX39B*	rs933208	6:31506648G > T	4.8	3.7	−1.1	Intron Retention; Cryptic Site Use
*CCT7*	rs1018342	2:73471653T > G	5.9	4.4	−1.5	Intron Retention
*PPIP5K2*	rs154290	5:102537200T > G	12.5	11.3	−1.3	Wildtype Only
*MYSM1*	rs232790	1:59131311G > T	10.4	8.7	−1.7	Wildtype Only
*PDGFRB*	rs246391	5:149497177T > C	6.2	3.6	−2.6	Wildtype Only
*AARS2*	rs324137	6:44273546A > C	10.2	8.9	−1.3	Wildtype Only
*USO1*	rs324726	4:76722353G > A	11.8	8.8	−3.0	Wildtype Only
*KIF13A*	rs624105	6:17855864G > C	14.1	13.0	−1.1	Wildtype Only
*TNFRSF1B*	rs653667	1:12251808T > G	3.7	2.4	−1.3	Wildtype Only
*CCDC93*	rs748767	2:118731573G > A	4.6	3.4	−1.1	Wildtype Only
*CAPN2*	rs751128	1:223951841T > C	5.3	4.2	−1.1	Wildtype Only
*FYCO1*	rs751552	3:46016851A > T	6.9	4.7	−2.2	Wildtype Only

^1^rsIDs are hyperlinked to their associated dbSNP page; ^2^If present, variant coordinates are hyperlinked to the ValidSpliceMut database; Thick bars separate SNP-affected exons with and without RNAseq-observed alternate splicing events.

Alternative mRNA splicing was observed in 14 SNPs: rs6467, rs36135, rs166062, rs171632, rs448580, rs469074, rs518928, rs694180, rs722442, rs752262, rs832567, rs909958, rs933208, rs1018342; **Table 3**). Reads spanning these regions revealed intron retention (N = 12), activation of cryptic splicing (N = 4), and complete exon skipping (N = 3). Eleven of these SNPs (79%) exhibited splicing patterns that significantly differed from the control alleles, and were therefore present in ValidSpliceMut. Interestingly, ValidSpliceMut contained entries for 7 of 10 SNPs where alternative splicing had not been found in the two patients reported in **Table 3** (rs246391, rs324137, rs624105, rs653667, rs748767, rs751128, rs751552). The observed significant splicing differences for these SNPs occurred in distinct tumor types, consistent with tissue-specific effects of these SNPs on splicing.

### Instances of Limited Corroboration of SNP-Related Predictions

Anticipated effects of the SNPs on splicing were not always confirmed by expression studies. Aside from incomplete or incorrect predictions, both design and execution of these studies as well as uncharacterized tissue specific effects could provide an explanation for these discrepancies. Furthermore, these undetected splicing events may have been targeted for NMD, however expression was not compared with mRNA levels from cells cultured with an inhibitor of protein translation. Stronger preexisting cryptic sites were, in some instances, not recognized nor was isoform abundance changed. These include: rs1893592 (6.4 and 5.2 bit cryptic donor sites 29 and 555 nt downstream of the affected donor); rs17002806 (a 5.7 bit site 67 nt downstream of the natural site); rs3747107 [creates a 4.9 bit cryptic site 2 nt downstream (observed by RNAseq; **Supplementary Image 1.8 D**)]; and rs2835585 (5.8 and 5.9 bit cryptic sites 60 and 87 nt upstream of the natural site). SNPs with modestly decreased natural site strength *(*0.2 to 4.5 bits) did not consistently result in exon skipping (for example, rs1893592, rs17002806, and rs2835655).

Six SNPs predicted to disrupt natural splice sites could not be confirmed. Splicing effects were not identified in the 4 SNPs where the information change was < 1 bit (<twofold). Genetic variability masked potential splicing effects of three of these SNPs, including rs16802, rs2252576, and rs8130564 (**Table 1**). PCR primer sets designed for *COL6A2* exon 21 (affected by rs17357592) did not produce the expected amplicon. Interpreting the results for rs16994182 (*CLDN14*) was complicated by the lack of a suitable internal reference. As *CLDN14* consists of three exons, any internal reference covering the affected second exon cannot parse whether differences in exon 2 expression were caused by the SNP or by general expression changes.

The T-allele rs2838010 was predicted to activate a donor splice site of a rare exon in IVS1 of *FAM3B* (GenBank Accession AJ409094). The cryptic pseudoexon was neither detected by RT-PCR nor expression microarray (**Supplementary Image 1.16**). Interestingly, this exon is expressed in a malignant lymphoma patient who is a carrier for this genotype [ICGC ID: DO27769; (**Supplementary Image 1.16C**)]. Although the T-allele is probably required to activate the pseudoexon, additional unknown splicing-related factors appear to be necessary.

## Discussion

Predicted SNP alleles that alter constitutive mRNA splicing are confirmed by expression data, and appear to be a common cause of alternative splicing. The preponderance of leaky splicing mutations and cryptic splice sites, which often produce both normal and mutant transcripts, is consistent with balancing selection (Nuzhdin et al., 2004) or possibly with mutant loci that contribute to multifactorial disease. Minor SNP alleles are often found in > 1% of populations (Janosíková et al., 2005). This would be consistent with a bias against finding mutations that abolish splice site recognition in dbSNP. Such mutations are more typical in rare Mendelian disorders (Rogan et al., 1998).

Exon-based expression microarrays and q-RT-PCR techniques were initially used to confirm the predicted impact of common and rare SNPs on splicing. Results were subsequently confirmed using RNAseq data for some of these SNPs (Dorman et al., 2014; Viner et al., 2014; Shirley et al., 2019). However, exon skipping due to rs1893592 was not consistently seen in all carriers. Although detected only in one type of tumor, this event may not be tissue specific, since five patients with the same genotype did not exhibit this isoform. Nevertheless, exon skipping was also observed in malignant lymphoma (**Supplementary Image 1.10D**). Intron retention in rs1805377 carriers was evident in only 22% of tumors. Increased total intron retention may be due to failure to recognize exons due to overlapping strong splice sites (Vockley et al., 2000; Rogan et al., 2003).

The splicing impacts of several of these SNPs have also been implicated in other studies. rs10190751 modulates the FLICE-inhibitory protein (c-FLIP) from its S-form to its R-form, with the latter having been linked to increased lymphoma risk (Ueffing et al., 2009). We observed the R-form to be twice as abundant for one of the rs10190751 alleles. Increased exon skipping attributed to rs1333973 has been reported in RNAseq analysis of *IFI44L* (Zhao et al., 2013a), which has been implicated in reduced antibody response to measles vaccine (Haralambieva et al., 2017). The splicing impact of *XRCC4* rs1805377 has been noted previously (Nalla and Rogan, 2005). This SNP has been implicated with an increased risk of gastric cancer (Chiu et al., 2008), pancreatic cancer (Ding and Li, 2015) and glioma (Zhao et al., 2013b). Similarly, the potential impact of rs1893592 in *UBASH3A* has been recognized (Kim et al., 2015) and is associated with arthritis (Liu et al., 2017) and type 1 diabetes (Ge and Concannon, 2018). Hiller et al. (2006) described the 3nt deletion caused by rs2243187 in *IL19* but did not report increased exon skipping. rs743920 was associated with change in *EMID1* expression (Ge et al., 2005), however its splicing impact was not recognized. Conversely, studies linking *TMPRSS3* variants to hearing loss did not report rs8130564 to be significant (Lee et al., 2013; Chung et al., 2014). Interestingly, rs2252576 (in which we did not find a splicing alteration) has been associated to Alzheimer’s dementia in Down syndrome (Mok et al., 2014).

rs2835585 significantly increased exon skipping in *TTC3*, however normal expression levels at the affected exon junction were not significantly altered. This was most likely due to the large difference in abundance between the constitutive and skipped splice isoforms (**Table 2**). The skipped isoform does not disrupt the reading frame and the affected coding region has not been assigned to any known protein domain (Tsukahara et al., 1996; Suizu et al., 2009). It is unclear whether allele-specific, exon skipping in this instance would impact TTC3 protein function or activity.

Why are so few natural splice sites strengthened by SNP-induced information changes? Most such changes would be thought to be neutral mutations, which are ultimately lost by chance (Fisher, 1930). Those variants which are retained are more likely to confer a selective advantage (Li, 1967). Indeed, the minor allele in rs2266988, which strengthens a donor splice site by 1.6 bits at the 5' end of the open reading frame in *PRAME* and occurs in 25% of the overall population (~50% in Europeans). Several instances of modest changes in splice site strength that would be expected to have little or no impact, in fact, alter the degree of exon skipping.

Allele frequency can significantly vary across different populations, which can be indicative of gene flow and migration of a population (Cavalli-Sforza and Bodmer, 1971) as well as, in the case of splicing variants, genetic load and fitness in a population (Rogan and Mucaki, 2011). The frequencies of several of the variants presented here are significantly different between ethnic and geographically defined populations (**Tables 1** and **3**). We examined allele frequencies of these variants in sub-populations in both HapMap and dbSNP version 153. For example, representation of the alleles of the *XRCC4* variant rs1805377 (where its A-allele leads to a 6 nt deletion of the gene's terminal exon) differs between Caucasians and Asians (for the G- and A-alleles, respectively). Different linkage disequilibrium patterns of this variant occur in Han Chinese (CHB) and Utah residents with Northern and Western European ancestry (CEU) populations (Zhao et al., 2013b). Similar differences in SNP population frequency include *EMID1* rs743920 (in HapMap: G-allele frequency is 47% in CHB, but only 7% in CEU and 10% in northern Swedish cohorts). This is consistent with dbSNP (version 153) where it is present in 72% in Vietnamese, but only 16% of a northern Sweden cohort). In *BACE2*, rs2252576, the T-allele is most prevalent at 84% in Yoruba in Ibadan, Nigeria populations (YRI), but only 8% in CHB. In *FCHSD1*, rs469074 the frequency of the G-allele is 37% in YRI and <1% in CHB. Some SNPs were exclusively present in a single population in the HapMap cohort (e.g. only the YRI population is polymorphic for *IL19* rs2243187, *WBP2NL* rs17002806, *DERL3* rs6003906, and *CLDN14* rs16994182).

Because of their effects on mRNA splicing, these differences in allele frequency would be expected to alter the relative abundance of certain protein isoforms in these populations. We speculate about whether isoform-specific representation among populations influences disease predisposition, other common phenotypic differences, or whether they are neutral. We suggest that SNPs decreasing constitutive splicing while increasing mRNA isoforms which alter the reading frame would be more likely to result in a distinct phenotype. q-RT-PCR experiments confirmed five SNPs which increased the fraction of mRNA splice forms causing a frameshift (**Table 1**), three of which simultaneously decrease constitutive splicing by ≥ 10-fold (*WBP2NL* rs17002806; *GUSBP11* rs3747107; and *IFI44L* rs1333973). rs3747107 and rs17002806 are much more common in YRI populations in HapMap (rs3747107 G-allele is present in 64% in YRI but only 23% in CEU; rs17002806 A-allele was not identified in any CHB or CEU individuals), while the A-allele of rs1333973 is much more common in CHB (76%, compared to 31% and 35% in CEU and YRI populations, respectively). These common variants are likely to change the function of these proteins and may influence individual phenotypes. A somewhat comprehensive catalog of DNA polymorphisms with splicing effects—confined or with increased prevalence in specific ethnic or geographically identifiable groups—could be derived from combining ValidSpliceMut with population-specific SNP databases. Aside from those phenotypes described earlier, genes implicated by GWAS or other analyses for specific disorders represent reasonable candidates for further detailed or replication studies aimed at identification of the risk alleles in these cohorts.

The extent to which SNP-related sequence variation accounts for the heterogeneity in mRNA transcript structures has been somewhat unappreciated, given the relatively high proportion of genes that exhibit tissue-specific alternative splicing (Pan et al., 2008; Wang et al., 2008; Baralle and Giudice, 2017). This and our previous study (Shirley et al., 2019) raise questions regarding the degree to which apparent alternative splicing is the result of genomic polymorphism rather than splicing regulation alone. Because much of the information required for splice site recognition resides within neighboring introns, it would be prudent to consider contributions from intronic and exonic polymorphism that produce structural mRNA variation, since these changes might be associated with disease or predisposition.

Individual information corresponds to a continuous molecular phenotypic measure that is well suited to the analysis of contributions of multiple, incompletely penetrant SNPs in different genes, as typically seen in genetically complex diseases (Cooper et al., 2013). Our protocol identifies low or nonpenetrant allele-specific alternative splicing events through bioinformatic analysis, and either q-RT-PCR, exon microarrays or RNAseq data analysis. Allele-specific splicing can also be determined by full-length alternative isoform analysis of RNA [or FLAIR (Workman et al., 2019)]. Differentiated splice forms are associated with specific alleles in heterozygotes with exonic SNPs. However, combining genome-based information with FLAIR may enable identification of intronic SNPs influencing splicing and low abundance alternative splice forms, which might otherwise be missed by FLAIR.

Targeted splicing analysis generally reproduces the results of our multi- genome-wide surveys of sequence variations affecting mRNA splicing. As splicing mutations and their effects were often observed in multiple tumor types, the impact of these mutations may be pleiotropic. Some events were only detected in q-RT-PCR data and not by RNAseq (and vice versa), highlighting the complementarity of these techniques for splicing mutation analyses. Results of this study increase confidence that the publicly (https://ValidSpliceMut.cytognomix.com) and commercially (https://MutationForecaster.com) available resources for information-theory based variant analysis and validation can distinguish mutations contributing to aberrant molecular phenotypes from allele-specific alternative splicing.

## Data Availability Statement

All datasets generated for this study are included in the article/**Supplementary Material**.

## Ethics Statement

Controlled-access TCGA and ICGC sequence data was approved by NCBI at the US National Institutes of Health (dbGaP Project #988: “Predicting common genetic variants that alter the splicing of human gene transcripts”; Approval Number #13930-11; PI: PK Rogan) and by the International Cancer Genome Consortium (ICGC Project #DACO-1056047; “Validation of mutations that alter gene expression”).

## Author Contributions

EM designed and performed all q-RT-PCR experiments, processed and analyzed publicly exon microarray data, and performed all formal analysis. BS performed data curation and software development. PR conceptualized the project and was the project administrator. EM and PR prepared the original draft of the manuscript, while EM, BS, and PR reviewed and edited the document.

## Conflict of Interest

PR founded and BS is an employee of CytoGnomix. The company holds intellectual property related to information theory-based mutation analysis and validation.

EM declares that the research was conducted in the absence of any commercial or financial relationships that could be construed as a potential conflict of interest.
