# Supplementary material for: Expression Changes Confirm Genomic Variants Predicted to Result in Allele-Specific, Alternative mRNA Splicing
Source: Front Genet. 2020 Mar 5;11:109. doi: 10.3389/fgene.2020.00109 (PMC7066660; doi:10.3389/fgene.2020.00109)
Supplement: Supplementary file 2 [file Table_1.docx]

| **Supplementary Table 1: q-RT-PCR Primers to Analyze Splice Forms**  This table lists all primers for all SYBR Green q-RT-PCR experiments described in this paper. The top four primer sets were simultaneously run with test primers to normalize sample-to-sample variation. Each group of primer sets which tested the effects of a specific mutation were designed to be similar in amplicon size and T_m_, though this was dependant on sequence. Primers designed to detect a specific splice form were placed over an exon junction (unless sequence of junction is detrimental to primer design, i.e. contains a G quartet, palindromic sequence). The tested splice form is described in the left-most column, while whether or not that splice form was detected is in the right-most column. Internal references are designed to amplify regions of the gene not expected to be affected by the SNP and is used to detect potential variation in the gene between individuals tested. The bolded regions of the primer sequence indicate the region of the primer that crosses the exon junction. Legend: F – Forward primer, R – Reverse primer, Acc. – Acceptor splice site. **^1^** When the mutation is located on the +1 or +2 positions of the splice site (within the exon), primers placed over the junction would lay on the polymorphism and therefore two primer sets are designed to account for this, i.e. *C21orf2* 5.7/2.4 bit cryptic donor (Exon 6 + 360nt Extension) G version with 100% match when tested mutation is a G. |
| --- |
| \| **Supplementary Table 1: q-RT-PCR Primers to Analyze Splice Forms** \| \| \| \| \| \| \| \| --- \| --- \| --- \| --- \| --- \| --- \| --- \| \| Splice Form Detection \| \| Size (nt) \| Tm  (ºC) \|  \| Sequence \| Detected \| \| External Reference \| \| \| \| \| \| \| \| *CCDC137* \| Exon 2-3  Junction \| 100 \| 60 \| F \| AGATTATGAGGAGCCGCCAAG \| Yes \| \| R \| GCTTCCTTCTCCAATGTCTTTCTG \| \| *DNAH1* \| Exon 43-44  Junction \| 104 \| 60 \| F \| CTGATGCGTCACTTCAACTACCTG \| Yes \| \| R \| CCAAGGAGTCCAT**CCAACCA** \| \| *FRMPD1* \| Exon 8-9 Junction \| 106 \| 59 \| L \| GAAGTATCGAGTACTTTGCACTGGC \| Yes \| \| R \| TCCTCCCTTTCTACCAC**CTGC** \| \| *VSP39* \| Exon 2-3 Junction \| 103 \| 60 \| L \| CTCAGTTCCATCAAAACTCG**GTTT** \| Yes \| \| R \| GAAGCCAAGACACACAGTGCTG \| \| Splice Form Detection Primers \| \| \| \| \| \| \| \| *XRCC4* rs1805377 (SYBR Green) \| \| \| \| \|  \| \| \| **11.5/3.9 bit upstream acc. (6nt included exon 8)** \| \| 106 \| 59 \| L \| AAAAGGAAAA**TTCTAGGCCTGATTCT** \| Yes \| \| R \| TCTGGGCTGCTGTTTCTCAGA \| \| **11.3/11.4 bit downstream acc. (6nt deleted exon 8)** \| \| 106 \| 59 \| L \| TTCAAGAAAAGGAAAA**GCCTGATT** \| Yes \| \| R \| TCTGGGCTGCTGTTTCTCAGA \| \| ***XRCC4* Internal Reference (exon 6-7)** \| \| 106 \| 59 \| L \| GGAAAGTGAAAACCAAACTGATCTCT \| Yes \| \| R \| TCTACTTGGTGCAATATCAGTGACATC \| \| *XRCC4* rs1805377 (dual labelled probe test) \| \| \| \| \| \| \| \| **Primers for Both Probes** \| \| - \| - \| L \| GAAATCTTGGGACAGAACCTAAA \| Yes \| \| R \| TCAGAGTTTCTAAAGACATGTTTTCA \| \| **Probe- upstream acc. (6nt included exon 8)** \| \| 141 \| 61 \| P \| AGCTTCAAGAAAAGGAAAA**TTCTAGGCCTG** \| Yes \| \| **Probe - downstream acc. (6nt deleted exon 8)** \| \| 135 \| 61 \| P \| CTTCAAGAAAAGGAAAA**GCCTGATTCTTCA** \| Yes \| \| *UBASH3A* rs1893592 (SYBR Green) \| \| \| \| \|  \| \| \| **9.1/4.3 bit natural donor (exon 10)** \| \| 107 \| 60 \| L \| TCCAGTCCAGAATTGCAG**GG** \| Yes \| \| R \| CAGGATGAGTTTGGCCGTCT \| \| **6.1 bit cryptic donor**  **(Exon 10 + 555nt Extension)** \| \| 107 \| 60 \| L \| ACTCAGAG**CCCTCCGCTGT** \| Yes \| \| R \| TCCAGGTTCCACTCGTATCTTGA \| \| **7.0 bit cryptic donor**  **(Exon 10+29nt extension)** \| \| 107 \| 60 \| L \| TTTGAGGACTGTCTAGTAGGAAAG**GG** \| Yes \| \| R \| GAGTTTGGCCGTCTGCACA \| \| **Exon 10 Skipping** \| \| 119 \| 65 \| L \| CCTTGCAG**GCTACCGTTGCAAG** \| No \| \| R \| TAGCGCGTC**CCCATCAGGAGT** \| \| ***UBASH3A* Internal Reference (Exon 8-9)** \| \| 107 \| 60 \| L \| TACAGGCCTTGCAG**GCTACC** \| Yes \| \| R \| AGTGGAGCATTGCTGCAGC \| \| *C21orf2* rs2070573 (SYBR Green) \| \| \| \| \|  \| \| \| **10.4 bit natural donor (exon 6)** \| \| 96 \| 62 \| L \| CCCAGGATGAACGTGGCCT \| Yes \| \| R \| GGACG**TTCCTGCCCCTGTG** \| \| **5.7/2.4 bit cryptic donor (Exon6+360nt Extension)**  **G version^1^** \| \| 96 \| 62 \| L \| GGGCAACAGGAGTCACGTGG \| Yes \| \| R \| GCAGTCAGGACGTT**CGGCA** \| \| **5.7/2.4 bit cryptic donor (Exon6+360nt Extension)**  **C version^1^** \| \| 106 \| 62 \| L \| TGAGGGCAACAGGAGTCACG \| Yes \| \| R \| CAGGATGGCAGTCAGGACGTT**A** \| \| ***C21ORF2* Internal Reference (Exon 4-5)** \| \| 96 \| 62 \| L \| TGGACAACCAGG**CTGTGACG** \| Yes \| \| R \| CGTGGCCTGTGCCCTCTCT \| \| *EMID1* rs743920 (SYBR Green) \| \| \| \| \|  \| \| \| **6.4 bit upstream acc. (6nt included exon 4)** \| \| 108 \| 59 \| L \| GAGCTGCGAGGAAG**TTGCAG** \| Yes \| \| R \| TTGAGACAACCTGAGAAGGCTGT \| \| **10.5/12.4 bit downstream acc. (6nt deleted exon 4)**  **C version^1^** \| \| 85 \| 59 \| L \| GTACAAGATAGTGACCGCCCGT \| Yes \| \| R \| CAAGGAGGCAGAGGAAC**CTTC** \| \| **10.5/12.4 bit downstream acc. (6nt deleted exon)**  **G version^1^** \| \| 85 \| 59 \| L \| GTACAAGATAGTGACCGCCCGT \| Yes \| \| R \| CAAGGAGGCAGAGGAAG**CTTC** \| \| ***EMID1* Internal Reference 1 (exon 4-5) (shorter amplicon)** \| \| 85 \| 59 \| L \| CTTCTCAG**GTTGTCTCAACTGCAG** \| Yes \| \| R \| AGTCAGCATGGTCAT**CTTGGC** \| \| ***EMID1* Internal Reference 2 (exon 4-5) (longer amplicon)** \| \| 111 \| 59 \| L \| CAGCCTTCTCAG**GTTGTCTCAAC** \| Yes \| \| R \| GAGGTACTGGCTGCTCTATGACAG \| \| *IL19* rs2243187 (SYBR Green) \| \| \| \| \|  \| \| \| **7.3/-0.3 bit upstream acc. (3nt included exon 5)** \| \| 101 \| 63 \| L \| AGCCAAACCCCAAAATCTTGAGAA \| Yes \| \| R \| GTGACACTGCCTCTGTTCCTG**ACAT** \| \| **7.6/7.5 bit downstream acc. (3nt excluded exon 5)** \| \| 101 \| 63 \| L \| CAGGAGCCAAACCCCAAAATCTT \| Yes \| \| R \| TGACACTGCCTCTGTTC**ACATTGC** \| \| **Exon 5 Skipping** \| \| 104 \| 63 \| L \| CAAGGATCATCAGGAGCCAAACC \| Yes \| \| R \| GGACCTCCAG**ACATTGCCGC** \| \| ***IL19* Internal Reference (Exon 2-4)** \| \| 102 \| 63 \| L \| AAAAGAGCCATC**CAAGCTAAGGACAC** \| Yes \| \| R \| GGTCACGCAGCACACATCTAAGG \| \| *PRAME* rs2266988 / rs2072049 (SYBR Green) \| \| \| \| \| \| \| \| **8.9/7.3 bit natural donor (exon 3) /**  **Internal Reference Primers for rs2072049** \| \| 101 \| 65 \| L \| TGAGACCTAGAAATCCAAGCGTTGGA \| Yes \| \| R \| GATGTATCGGCTCTGAATGGAACC**C** \| \| **Exon 3 Skipping** \| \| 95 \| 65 \| L \| TGGTGAACTCTCTGAGGAAAAAC**GGT** \| Yes \| \| R \| CCCTGCCAGCTCCACAAGTCTC \| \| ***PRAME* Internal Reference (Exon 5-6) /**  **Test primers for rs2072049** \| \| 101 \| 65 \| L \| TCAGTTGCTCAG**GCACGTGATGA** \| Yes \| \| R \| CGCTGGGACTCTGGGACAGATG \| \| *TTC3* rs2835585 (SYBR Green) \| \| \| \| \|  \| \| \| **6.4/4.4 bit natural acc. (exon 3)** \| \| 102 \| 64 \| L \| TTATGTTCGTGTGACTCAGCTTTACTGTGA \| Yes \| \| R \| CATATACTGCAGATGTCAAATT**CCAAATTCC** \| \| **Exon 3 Skipping** \| \| 102 \| 64 \| L \| ATGATTATGTTCGTGTGACTCAGCTTTACTG \| Yes \| \| R \| TCAGAAGCACGTGAATTG**CCAAA** \| \| **Intron 2 (IVS2) Inclusion** \| \| 101 \| 60 \| L \| GGGTG**GGTGTGCAATATAAAGATT** \| Yes \| \| R \| CCTCCCTTTGTAGTCTTTCCATAAATTAA \| \| **6.9 bit cryptic acceptor (exon 3 + 60 nt)** \| \| 105 \| 64 \| L \| TTATGTTCGTGTGACTCAGCTTTACTGTGA \| No \| \| R \| GCCTCATTTTTTTAATGAATCAACA**CCAA** \| \| **7.2 bit cryptic acceptor (exon 3 + 87 nt)** \| \| 107 \| 64 \| L \| TCGTGTGACTCAGCTTTACTGTGATGG \| No \| \| R \| TCAACACTTAATAGTAAAAAGGCAAAAATGGAT**C** \| \| ***TTC3* Internal Reference (Exon 4-6)** \| \| 102 \| 64 \| L \| GATGGAAGATATTGTGGATTTGGCAAAG \| Yes \| \| R \| GCCAAGATTTT**ATTTTCTATTTTACAACCAATTCTC** \| \| *TTC3* rs2835655 (SYBR Green) \| \| \| \| \|  \| \| \| **Natural Exon 38-39 Junction (unaffected)** \| \| 113 \| 64 \| L \| GTACTTGAAAACTGGAAGGAGAGTGAAGTGTATAAG \| Yes \| \| R \| GGATATGCTGCAGGATCA**CGGCTA** \| \| **Exon 39 Skipping** \| \| 113 \| 65 \| L \| AAAACTGGAAGGAGAGTGAAGTGTATAAGCTACAG \| Yes \| \| R \| CCTTAATTTGTTCTTCAAACTGAGA**CGGC** \| \| ***TTC* Internal Reference (Exon 40-41)** \| \| 113 \| 65 \| L \| AGAACAAATTAAGGCAATTAAAAATGGTTCTCG \| Yes \| \| R \| CAGGGAGTAACTCGGGATGAAC**CGT** \| \| *FAM3B* rs2838010 (SYBR Green) \| \| \| \| \|  \| \| \| **Natural Exon 1-2 Junction (unaffected)** \| \| 95 \| 59 \| L \| CTGGTG**GCCTGCTCAAGGT** \| Yes \| \| R \| GGGTGCATCTGGAATGAGCT \| \| **1.4/9.2 bit donor of cryptic exon within IVS1** \| \| 93 \| 59 \| L \| CCATTGGCTGGTG**GTTCAC** \| No \| \| R \| TTAACTTAACCAACAAACGTCCTTGA \| \| ***FAM3B* Internal Reference (exon 3-5)** \| \| 96 \| 59 \| L \| GCCCATCTGACACCTATGCC \| Yes \| \| R \| GTTCTCCCATAAGT**AGGTTATCCTCAA** \| \| *WBP2NL* rs17002806 (SYBR Green) \| \| \| \| \|  \| \| \| **10/6.5 bit natural donor (exon 6)** \| \| 90 \| 60 \| L \| AGGATAAGGAGGACGACTCAG**CTT** \| Yes \| \| R \| CTGTCCTTGAGAACATGGGAGC \| \| **Exon 5-6 Junction (unaffected)** \| \| 109 \| 60 \| L \| TTTCCACTTAGAACCTTAAATGACTGGT \| Yes \| \| R \| CATAGACAATAA**CTGAACAAGGCATCTG** \| \| **Exon 6 Skipping** \| \| 101 \| 60 \| L \| AAATGACTGGTTCAGCTCTATGGG \| No \| \| R \| AGATCAATCAATGACTCTAAG**CTGAACA** \| \| **6.6 bit cryptic donor (exon 3 + 25 nt)** \| \| 101 \| 59 \| L \| AGACTCACCAAGCAAAGAGGTACC \| Yes \| \| R \| CAATCAATGACTCTAAG**CTGCGAG** \| \| **5.7 bit cryptic donor (exon 3 + 67 nt)** \| \| 101 \| 60 \| L \| GGAGGACGACTCAGGTATGTGATC \| No \| \| R \| GATCAATCAATGACTCTAAG**CTTCCC** \| \| ***WBP2NL* Internal Reference (exon 4-5)** \| \| 106 \| 60 \| L \| TGGTGAAAGCTGCCTCTGCT \| Yes \| \| R \| CACATATTCCCTTCCCCAGTAATTAC \| \| *IFI44L* rs1333973 (SYBR Green) \| \| \| \| \|  \| \| \| **9.5/5.0 bit natural acceptor (exon 2)** \| \| 94 \| 59 \| L \| CCGTGGCTGCTCGATAAATC \| Yes \| \| R \| TTGTCACTTCCATTGTTCTATAT**CTGTTT** \| \| **Exon 2 Skipping** \| \| 94 \| 59 \| L \| AACCGTGGCTGCTCGATAAA \| Yes \| \| R \| CGTCTAGGTTATCCTTAATTC**CTGTTTC** \| \| ***IFI44L* Internal Reference (exon 5-6)** \| \| 94 \| 59 \| L \| TGTATGCCAGACAGATATCAG**TTTAATTC** \| Yes \| \| R \| GAATCCTGTCCTTCAGAGATGGAG \| \| *CFLAR* rs10190751 (SYBR Green) \| \| \| \| \|  \| \| \| **17.4/9.9 bit acceptor of upstream exon 7 (s form)** \| \| 93 \| 59 \| L \| AGCAGGGACAAGTTACAGGAATGT \| Yes \| \| R \| GCATAGGGTGTTATCAT**CCTGAAGT** \| \| **6.9 bit acceptor of downstream exon 7** \| \| 94 \| 59 \| L \| CAAGGAGCAGGGACAAGTTACAG \| Yes \| \| R \| TCCCATTATGGAG**CCTGAAGTT** \| \| ***CFLAR* Internal Reference (exon 3-4)** \| \| 91 \| 59 \| L \| AATCTGATGTGTCCTCATTAATTTTCC \| Yes \| \| R \| ACCACAAGGTCCAAGAAACT**CTTC** \| \| *LPP* rs13076750 (SYBR Green) \| \| \| \| \|  \| \| \| **9.3/-1.6 bit upstream acc. (7nt included exon 1a)** \| \| 91 \| 60 \| L \| TTCCCTGTGTTCTGCTTTTTTCAT \| Yes \| \| R \| CCAACTGCAATGCTAGTGT**CAATT** \| \| **2.0 bit downstream acc. (7nt excluded exon 1a)** \| \| 89 \| 60 \| L \| TGATTCCCTGTGTTCTGCTTTTTT \| Yes \| \| R \| TCAGCCAACTGCAATG**CAAT** \| \| **Exon 1a Skipping (cryptic exon in IVS1)** \| \| 89 \| 60 \| L \| TCCCTGTGTTCTGCTTTTTTCAT \| Yes \| \| R \| GGGTGAGACATTGTTGGAAT**CAAT** \| \| ***LPP* Internal Reference (exon 3-4)** \| \| 90 \| 60 \| L \| CTGGAGGTGAGG**GTGATTTTCTT** \| Yes \| \| R \| GAGGAGGAAAGTTTCCAGAGATAGATG \| \| *GUSBP11* rs3747107 (SYBR Green) \| \| \| \| \|  \| \| \| **Upstream Exon 12 (8.7/1.4 bit acc.)** \| \| 71 \| 59 \| L \| TCATGACTAACCAGT**AGTGGGTGC** \| Yes \| \| R \| CTGGTCGTAGGCTGGATGTGT \| \| **1.6/7.5 bit upstream cryptic Acc. (-2 nt)** \| \| 69 \| 59 \| L \| TCATGACTAACCAGT**TGGGTGC** \| No \| \| R \| CTGGTCGTAGGCTGGATGTGT \| \| **8.3 bit upstream cryptic acc. (-114 nt)** \| \| 76 \| 58 \| L \| CCGATTTCATGACTAACCAGT**GAT** \| Yes \| \| R \| AGAGTCTCCTTGGGAAACTTACCA \| \| **10.9 bit upstream cryptic acc. (-118 nt)** \| \| 76 \| 59 \| L \| TTTCATGACTAACCAGT**TTAGGATGG** \| Yes \| \| R \| AGAGTCTCCTTGGGAAACTTACCA \| \| **6.0 bit upstream cryptic acc. (-156 nt)** \| \| 69 \| 59 \| L \| GCCGATTTCATGACTAACCAGT**GT** \| No \| \| R \| CCATCCTAACTGGGAAGACAAAAAG \| \| **Downstream Exon 12 (8.9 bit acc.)** \| \| 70 \| 59 \| L \| CCGATTTCATGACTAACCAGT**GTC** \| Yes \| \| R \| TCACAGGACGGCAGGAACA \| \| ***GUSBP11*** **Internal Reference (exon 10-11)** \| \| 69 \| 59 \| L \| GTGGACATTGACCCCACTGG \| Yes \| \| R \| GAACATCAGAGGTGGATC**CTGC** \| \| *DERL* 3 rs6003906 (SYBR Green) \| \| \| \| \|  \| \| \| **2.2/0.3 bit natural acceptor (exon 5)** \| \| 92 \| 59 \| L \| ATGCTGGAAGAGGGCTCCTT \| Yes \| \| R \| AGGAGTCCCAGCAG**GGTCA** \| \| **11.3 bit downstream alt. acc. (exon 5 – 123nt) Uses extended exon 4** \| \| 95 \| 59 \| L \| GAGGGCCCACTCTGTGCTC \| Yes \| \| R \| ACGGTGC**CTGGCTCTTTTG** \| \| **11.3 bit downstream alt. acc. (exon 5 – 123nt) Uses short exon 4** \| \| 89 \| 59 \| L \| ATGCTGGAAGAGGGCTCCTT \| No \| \| R \| AGGAACGGTGC**GGTCATAAG** \| \| ***DERL3* Internal Reference (exon 2-3)** \| \| 77 \| 59 \| L \| GGAGCTCCTCAGCCCCTTT \| Yes \| \| R \| TGACGAGCCTCCAGAC**CTG** \| \| *ARFGAP3* rs1018448 (SYBR Green) \| \| \| \| \|  \| \| \| **10.6/12.8 bit natural acceptor (exon 12)** \| \| 100 \| 59 \| L \| AATCACCCATTATGGCAAAACC \| Yes \| \| R \| AACTCCACTGGCTCGTCAAAGT \| \| **5nt upstream acc. (exon 12)**  *** Potentially misaligned** \| \| 101 \| 59 \| L \| CAGGAATCACCCATTATGGCA \| No \| \| R \| TTAACTCCACTGGCTCGTCAA**CT** \| \| **Exon 12 Skipping** \| \| 100 \| 60 \| L \| ACCATAGAGCAGGAATCACCCAT \| Yes \| \| R \| GAGCAGTAGGT**CTTGAGCTGGAAG** \| \| ***ARFGAP3* Internal Reference (exon 13-14)** \| \| 100 \| 58 / 60 \| L \| ATACAGATGAGGCCCAGAAGAAGTT \| Yes \| \| R \| CGGGCCCTGGTCTCATA**AT** \| \| *BCR* rs16802 (SYBR Green) \| \| \| \| \|  \| \| \| **8.8/9.4 bit natural acceptor (exon 14)** \| \| 91 \| 59 \| L \| GCTGCAGATGCTGACCAACTC \| Yes \| \| R \| CCCCGGAGACTCATCAT**CTTC** \| \| ***BCR* Internal Reference (exon 10-11)** \| \| 93 \| 59 \| L \| ATCCAGAGAGAGAAG**AGGGCG** \| Yes \| \| R \| CATAAGCAGCAGCAGTGACTCC \| \| *BACE2* rs2252576 (SYBR Green) \| \| \| \| \|  \| \| \| **9.0/9.6 natural acceptor (exon 5)** \| \| 97 \| 60 \| L \| CTTAATCTGGACTGCAGAGAG**TATAACG** \| Yes \| \| R \| CCACCGCATCAAACACCTTC \| \| ***BACE* Internal Reference (exon 3-4)** \| \| 97 \| 60 \| L \| TTAAATGGAATGGAATACTTGGCCT \| Yes \| \| R \| TTTGCTTGTGTCACCAGGGA \| \| *TMPRSS3* rs8130564 (SYBR Green) \| \| \| \| \|  \| \| \| **6.8/6.3 natural acceptor (exon 6)** \| \| 103 \| 59 \| L \| CATGTGCTCCGATGACTGGA \| Yes \| \| R \| CAGCGAGCTCACTCTGAGGTTAT \| \| ***TMPRSS3* Internal Reference (exon 7-8)** \| \| 103 \| 59 \| L \| GTGCACAG**CCTGTGGTCATAGA** \| Yes \| \| R \| CTGGAACTGAAGGCTGGCC \| \| *CLDN14* rs16994182(SYBR Green) \| \| \| \| \|  \| \| \| **Natural Exon 2-3 Junction** \| \| 94 \| 63 \| L \| CTTTCAGATATAGCACTGGACTTGGCTG \| Yes \| \| R \| ACAGTTCTTGACTTCTTGGCTTGTGTC \| \| **Exon 2 Skipping** \| \| 95 \| 63 \| L \| CTGCCAAGAGAGGGAGTAAGATGTTCA \| Yes \| \| R \| GGAGCC**CGTGCTGCTGTGT** \| |

|  |
| --- |
