# Supplementary material for: Expression Changes Confirm Genomic Variants Predicted to Result in Allele-Specific, Alternative mRNA Splicing
Source: Front Genet. 2020 Mar 5;11:109. doi: 10.3389/fgene.2020.00109 (PMC7066660; doi:10.3389/fgene.2020.00109)
Supplement: Supplementary file 3 [file Table_2.docx]

| **Supplementary Table 2: SNPs in ValidSpliceMut Splicing Mutation Database** | | | | |
| --- | --- | --- | --- | --- |
| **Gene** | **rsID** | **Variant^1^** | **# Cases in ValidSpliceMut** | **RNAseq Evidence Type (Veridical Results)**^2^ |
| *C21orf2* | rs2070573 | [21:45750346C>A](https://validsplicemut.cytognomix.com/view.php?targets=191610+812565+191609+79880+191608) | 5 | Intron Inclusion (Junction Spanning): $\bar{p}$ = 0.024 (3/5)  Intron Inclusion (Read Abundance): $\bar{p}$ = 0.019 (5/5) |
| *CFLAR* | rs10190751 | [2:202006096G>A](https://validsplicemut.cytognomix.com/view.php?targets=791453+554189+554190+554193+554194+791452+791454+554187+791455+813970+813971+813972+813973+917749+554188+554186+73687+428256+79783+428255+428257+428258+428259+428260+428261+428262+428263+428264+917750) | 29 | Intron Inclusion (Junction Spanning): $\bar{p}$ = 0.009 (13/29)  Intron Inclusion (Junction Spanning with mutation):  $\bar{p}$ = 0.006 (28/29)  Intron Inclusion (Read Abundance): $\bar{p}$ = 0.009 (6/29) |
| *LPP* | rs13076750 | [3:188059443A>G](https://validsplicemut.cytognomix.com/view.php?targets=349138+349139+633447+633448+797460+797461+922409+922410)^3^ | 8 | Intron Inclusion (Junction Spanning): $\bar{p}$ = 0.007 (2/8)  Intron Inclusion (Junction Spanning with mutation):  $\bar{p}$ = 0.0001 (2/8)  Intron Inclusion (Read Abundance): $\bar{p}$ = 0.009 (1/8) |
| *PRAME* | rs2072049 | [22:22891081G>T](https://stagedvalidsplicemut.cytognomix.com/view.php?targets=80564+427772+427773) | 3 | Intron Inclusion (Junction Spanning): $\bar{p}$ = 0.028 (2/3)  Intron Inclusion (Read Abundance): $\bar{p}$ = 0.01 (1/3) |
| *TTC3* | rs2835585 | [21:38460488 T>A](https://validsplicemut.cytognomix.com/view.php?targets=79127&referenceName=21&alternateBases=A&start=38460488&referenceBases=T&assemblyId=GRCh37&includeDatasetResponses=HIT) | 1 | Intron Inclusion (Read Abundance): $\bar{p}$ = 0.05 |
| *UBASH3A* | rs1893592 | [21:43855067A>C](https://validsplicemut.cytognomix.com/view.php?targets=426812+426813+426814+426815+426816+426817+426818+426819+447351) | 9 | Exon Skipping (Junction Spanning): $\bar{p}$ = 0.0000 (3/9)  Intron Inclusion (Junction Spanning): $\bar{p}$ = 0.0003 (6/9)  Intron Inclusion (Junction Spanning with mutation):  $\bar{p}$ = 0.002 (9/9)  Intron Inclusion (Read Abundance): $\bar{p}$ = 0.0000 (7/9) |
| *XRCC4* | rs1805377 | [5:82648943G>A](https://validsplicemut.cytognomix.com/view.php?targets=907750+898346+898347+898348+898349+907749+933253+907751+933252+898344+933254+933255+933256+933257+898345+819965+898343+191626+78198+191621+191622+191623+191624+191625+191627+898342+813246+813247+813248+819964+898341+933258) | 32 | Intron Inclusion (Junction Spanning): $\bar{p}$ = 0.023 (3/32)  Intron Inclusion (Junction Spanning with mutation):  $\bar{p}$ = 0.0000 (26/32)  Intron Inclusion (Read Abundance): $\bar{p}$ = 0.014 (7/32) |
| ^1^ Variant (hg19 coordinates) with hyperlinked to ValidSpliceMut. ^2^ Only significant cases computed in average p-value. ^3^ *LPP* exon affected is rarely used and is categorized in ValidSpliceMut as a cryptic site splicing change. As the software did not consider the affected exon, any exon skipping reads would be instead for the nearest associated exon, which explains why exon skipping is not flagged in ValidSpliceMut despite its considerable increase detected by qRT-PCR. | | | | |
